# Cognate Interaction With CD4^+^ T Cells Instructs Tumor-Associated Macrophages to Acquire M1-Like Phenotype

**DOI:** 10.3389/fimmu.2019.00219

**Published:** 2019-02-22

**Authors:** David Eisel, Krishna Das, Elke Dickes, Rainer König, Wolfram Osen, Stefan B. Eichmüller

**Affiliations:** ^1^GMP & T Cell Therapy Unit, German Cancer Research Center (DKFZ), Heidelberg, Germany; ^2^Biosciences Faculty, University of Heidelberg, Heidelberg, Germany; ^3^Biopharmaceutical New Technologies (BioNTech) Corporation, Mainz, Germany; ^4^Division of Virology, Innsbruck Medical University, Innsbruck, Austria; ^5^Integrated Research and Treatment Center for Sepsis Control and Care, Jena University Hospital, Jena, Germany

**Keywords:** tumor associated macrophages, CD4^+^ T cells, tumor microenvironment, adoptive T cell transfer, M2 macrophage, T cell therapy, CD206, iNOS

## Abstract

The immunosuppressive tumor microenvironment (TME) established by tumor cells, stromal cells and inhibitory immune cells counteracts the function of tumor reactive T cells. Tumor associated macrophages (TAMs) showing functional plasticity contribute to this process as so called M2-like macrophages can suppress the function of effector T cells and promote their differentiation into regulatory T cells (Tregs). Furthermore, tumor antigen specific CD4^+^ T effector cells can essentially sustain anti-tumoral immune responses as shown for various tumor entities, thus suggesting that cognate interaction between tumor antigen-specific CD4^+^ Th1 cells and TAMs might shift the intra-tumoral M1/M2 ratio toward M1. This study demonstrates repolarization of M2-like PECs upon MHC II-restricted interaction with tumor specific CD4^+^ Th1 cells *in vitro* as shown by extensive gene and protein expression analyses. Moreover, adoptive transfer of OVA-specific OT-II cells into C57BL/6 mice bearing OVA expressing IA^b−/−^ tumors resulted in increased accumulation of M1-like TAMs with enhanced M1 associated gene and protein expression profiles. Thus, this paper highlights a so far underestimated function of the CD4^+^ Th1/TAM axis in re-conditioning the immunosuppressive tumor microenvironment.

## Introduction

Within the cellular immune system, CD8^+^ cytotoxic T cells (CTLs) have been considered as the central effector cells in tumor immune surveillance, due to their capacity of direct tumor cell killing (1). However, it has become clear that other cell types such as NK cells (2) or activated macrophages (3) are also endowed with cytotoxic function, thereby contributing to tumor cell eradication. Even CD4^+^ T cells were shown to acquire cytotoxic capacity against established tumors under certain circumstances (4, 5). However, tumors are often infiltrated by inhibitory immune cells such as myeloid derived suppressor cells (MDSCs) or tumor associated macrophages (TAMs) acting in concert with regulatory CD4^+^ T cells to create an immunosuppressive microenvironment that becomes hostile to activated T effector cells (6, 7). Interestingly, TAMs play a central role in this scenario due to their marked functional plasticity, allowing them to shift between various functional phenotypes, ranging from M2-like macrophages with immunosuppressive function to inflammatory M1-like macrophages equipped with immunostimulatory capacity (8). Consequently, at early tumor stages, when no suppressive milieu has been established yet, many tumors harbor predominantly M1-like macrophages (9), whereas M2-like TAMs tend to accumulate within the hypoxic areas of late stage tumors (10). Functional polarization of TAMs can be driven by soluble factors present in the tumor microenvironment. Thus, as reviewed by Mantovani et al. tumor cell derived cytokines like TGF-ß or CSF-1 as well as metabolic compounds (i.e., lactic acid) released by tumor cells can drive M2 polarization of TAMs. Similarly, IL-4 and IL-13 secreted by tumor resident Th2 cells or IL-10 released by Treg and B cells induce M2-like TAM differentiation. Moreover, stimulation of TAMs by immune complexes can result in M2 polarization. On the other hand, Th1 responses have been described to promote M1 differentiation in early tumor stages through secretion of IFNγ (11, 12).

High TAM numbers have been generally associated with poor prognosis (13), although exceptions to this rule do exist (14). Thus, therapeutic targeting of TAMs by depletion or reprogramming represents an obvious strategy for cancer treatment and has been investigated in a number of studies (7, 15). However, TAM targeting should ensure selective hit of M2-like TAMs, as M1-like macrophages can be essential for successful immunotherapy (16, 17). Selective repolarization of M2-like TAMs thus provides an option of neutralizing M2-associated activity without harming M1-like macrophages. In fact, cognate interaction between CD4^+^ Th1 cells and macrophages can drive M1 polarization, thereby offering an opportunity to achieve this goal. Thus, peptide loaded human PECs polarized by IL-6 and PGE2 were shown to acquire M1-like phenotype upon co-culture with a HPV E7 specific CD4^+^ T cell clone (18). Similarly, TAMs isolated from tumors of a murine multiple myeloma model became activated when co-cultured with TCR transgenic CD4^+^ Th1 cells specific for the tumor antigen (19, 20). However, to our knowledge, no results have been published so far that focused on the cognate interaction between tumor-associated macrophages and CD4^+^ Th1 cells in a wildtype *in vivo* situation. In light of established protocols using IL-4 (21–23) or IL-13 (23) to induce M2 polarization among isolated macrophages *in vitro*, we applied IL-4 as polarizing agent to obtain M2-like macrophages as starting population for co-culture experiments with CD4^+^ Th1 cells. Applying extensive gene and protein expression analyses as well as functional assays, we demonstrate that MHC II restricted interaction with CD4^+^ Th1 cells instructs PEC-derived M2-like macrophages to acquire M1-like phenotype and function. Moreover, we demonstrate that adoptive transfer of tumor antigen specific CD4^+^ Th1 cells into tumor bearing C57BL/6 mice results in accumulation of TAMs with a M1 shifted phenotype and enhanced M1-associated gene expression. These results may thus highlight a so far underestimated function of tumor antigen specific CD4^+^ Th1 cells in the context of T cell mediated tumor attack.

## Materials and Methods

A list of abbreviations is given in the Supplementary Materials.

### Isolation, Purification, and Polarization of PECs

Animal experiments were approved by District Government in Karlsruhe, Germany (approval ID 35–9158.81/G-211/16). C57BL/6 mice (Charles River, Sulzfeld, Germany) were injected intraperitoneally (i.p.) with 1 ml of 3 % (w/v) thioglycollate solution (Applichem, Darmstadt, Germany). Four days later, mice were sacrificed and injected with 8 ml of ice cold PBS (Thermo Fisher Scientific, Waltham, USA) into the peritoneal cavity. Peritoneal fluid containing PECs was aspirated and centrifuged at 300 × g for 10 min. PECs were resuspended in DMEM (Thermo Fisher Scientific) containing 10 % FBS (Biochrom, Darmstadt, Germany) and 2 × 10^6^ or 0.5 × 10^6^ cells were seeded into each well of a 6 or 24 well plate (TPP, Trasadingen, Switzerland), respectively. After 2 h, the medium was removed and adherent cells were washed three times with PBS. In order to induce M2 or M1 polarization, cells were cultured in DMEM supplemented with either 10 ng/ml IL-4 (BioLegend, San Diego, USA) or a combination of 100 ng/ml LPS (Sigma-Aldrich, St. Louis, USA) and 50 ng/ml IFNγ (Thermo Fisher Scientific), respectively, for 4, 24, 48, or 72 h. For subsequent analysis, cells were detached with 500 μl Accutase (Merck, Darmstadt, Germany) at 37°C for 5 min and harvested using a cell scraper.

### Lentiviral Transduction of B16F10/IA^b-/-^ Cells

The B16F10 derived IA^b^ knock-out clone B16F10/M2KO established previously (24) was used to generate an ovalbumin (OVA) expressing knock-out clone lacking IA^b^ expression. The transduction of tumor cell lines using retroviral particles was performed by the Genomics and Proteomics Core Facility of the German Cancer Research Center. The OVA encoding nucleotide sequence (RefSeq NM_205152.2.) flanked by attL recombination sites was synthesized and cloned into a pMX plasmid (Thermo Fisher Scientific, Waltham, USA). The sequences were shuttled into lentiviral expression vectors adding a C-terminal IRES sequence coupled to a neomycin resistance gene by gateway recombination. For the generation of lentiviral particles, HEK293FT cells (Thermo Fisher Scientific, Waltham, USA; Cat. No. R70007) were transduced with the lentiviral OVA expression constructs and transfected with 2nd generation viral packaging plasmids VSV.G (Addgene, plasmid #14888, MiIdlesex, UK) and psPAX2 (Addgene, plasmid #12260). Two days later, virus containing supernatants were collected and cleared by centrifugation (500 × g for 5 min). Supernatants were passed through a 0.45 μm filter to remove remaining cellular debris and B16F10 IA^b^ knockout cell layers showing 70% confluency were transduced with viral particles in the presence of 10 μg/ml polybrene (Merck KGaA, Darmstadt, Germany; Cat. No. TR-1003-G). The virus containing medium was replaced by selection medium containing 1 mg/ml Geneticin (Thermo Fisher Scientific) 1 day post transduction. After 2 weeks, clones were picked and expanded. The clone used in this study is designated B16F10/M2KO/OVA.

### Generation of an OVA-Specific CD4^+^ Th1 Cell Line

C57BL/6 mice were immunized subcutaneously (s.c.) with 100 μg of IA^b^ restricted OVA peptide ISQAVHAAHAEINEAGR (aa 323–339) emulsified 1:1 in complete Freund's Adjuvant (Sigma-Aldrich). After 7 days, mice were sacrificed and 6 × 10^6^ splenocytes were cultured in Minimum Essential Medium Eagle Alpha Modification (Sigma-Aldrich) supplemented with 2 μg/ml peptide, 2 mM L-Glutamine (Thermo Fisher Scientific), 10% FCS, 50 μM beta-Mercaptoethanol (Sigma-Aldrich), 100 U/ml Penicillin and 100 μg/ml Streptomycin (Sigma-Aldrich). Every 7 days, half of the supernatant was exchanged by the culture medium mentioned above, supplemented with 12.5 mM Methyl α-D-mannopyranoside (Sigma-Aldrich) and 2.5 % (v/v) culture supernatant from conA stimulated rat spleen cells, as a source of interleukin-2 (complete T cell medium). Spleen cell cultures were restimulated every 4 weeks by the addition of 6 x 10^6^ irradiated syngeneic feeder cells together with antigenic peptide (2 μg/ml). This T cell line showed specific IFNγ release upon co-culture with IA^b^ positive target cells pulsed with the OVA-specific T cell epitope aa323-339 and is thus considered as Th1 biased CD4^+^ T cell line.

### Peptide Loading of PECs

Polarized PECs were incubated at 37°C for 45 min in X-VIVO™ 20 serum free medium (Lonza Group, Baser, Switzerland) containing 1 μg/ml IA^b^ restricted OVA specific peptide (aa 323–339) or HBV core antigen derived peptide (aa 128–140) as control. Cells were washed 3–5 times with PBS to remove unbound peptides.

### Co-culture of PECs and OVA Specific CD4^+^ Th1 Cells

M2 polarized PECs (24 h) were loaded with peptide (5 μg/ml) and co-cultured with OVA specific CD4^+^ Th1 cells for 24 h (ratio 4:1) in the absence of IL-4. Supernatants were collected to measure IFNγ concentrations and macrophages were washed three times to remove the non-adherent T cells. The cells were subsequently used for further analysis.

### IFN**γ** ELISpot Assays

IFNγ ELISpot assays were performed using Multiscreen ELISpot plates (Merck) coated with 1 μg/ml goat anti-mouse IFNγ capture antibody (Becton Dickinson, Franklin Lakes, USA) overnight at 4°C. After blocking with serum containing medium, graded numbers of OVA-specific T cells were added to 5 × 10^4^ peptide loaded PECs in a total volume of 200 μl per well and cells were co-cultured for 16–18 h. On the next day, cells were incubated with 2 μg/ml biotinylated rat anti-mouse IFNγ antibody (Becton Dickinson) for 1 h followed by incubation with avidin-conjugated alkaline phosphatase (Becton Dickinson) for 30 min. IFNγ-specific spots were visualized by addition of BCIP/NBT (Sigma-Aldrich) and the reaction was stopped with distilled water. Spots were counted using an ELISpot reader (AID, Straßberg, Germany). All antibodies used for the ELISpot Assay are depicted in Table S1.

### Immunofluorescence Staining and Flow Cytometry

Immunofluorescence staining was performed using monoclonal antibodies shown in Table S2. As controls, the respective isotype matched antibodies against irrelevant epitopes were included. Cells (2 × 10^5^) were incubated at 4°C for 20 min in a mixture of rat anti-mouse CD16/CD32 (Becton Dickinson), normal Syrian hamster serum (Jackson Laboratory, Bar Harbor, USA) and rat serum (GeneTex, Irvine, USA) in a total volume of 100 μl to block Fc-receptors. Subsequently, cells were incubated with fluorochrome conjugated antibodies diluted in PBS containing 3 % FCS and LIVE/DEAD Fixable Yellow Dead Cell dye (1:1000) at 4°C for 1 h. In case of intracellular staining cells were fixed and permeabilized using FoxP3 staining Kit (eBioscience, Waltham, USA) according to manufacturer's instructions. Thereafter, the cells were incubated with fluorochrome conjugated antibodies diluted in Permeabilisation Buffer (Thermo Fisher Scientific) at 4°C for 1 h. Finally, cells were analyzed with a FACSCanto II or LSR II (Becton Dickinson) cytometer or sorted using a FACS Aria I or II and data were evaluated with FlowJo software (Version 10).

### RNA Isolation and Quantitative Real-Time PCR

RNA of primary macrophages sorted from tumor tissue was isolated using the RNeasy Micro Kit according to manufacturer's instructions. To isolate RNA from PECs, the cells were washed twice with PBS, lysed by the addition of 1 ml QIAzol Lysis Reagent (Qiagen, Venlo, Netherlands) per well and transferred into a 1.5 ml Eppendorf tube. After the addition of 200 μl chloroform and subsequent centrifugation, the aqueous phase was transferred into a fresh tube containing 600 μl 75% Ethanol (Sigma-Aldrich). RNA extraction was carried out using RNeasy columns according to manufacturer's instructions. RNA isolated from PECs was subjected to reverse transcription using Transcriptor First Strand cDNA Synthesis Kit (Hoffmann-La Roche, Basel, Switzerland). RNA extracted from primary macrophages was reverse transcribed using the iScript cDNA Synthesis Kit (Bio-Rad Laboratories, Hercules, USA). Gene expression was quantified using quantitative real-time PCR. Therefore, 2 X Power SYBR® Green PCR Master Mix (Thermo Fisher Scientific), 10 ng cDNA, 400 nM of each primer pair (200 nM each) and nuclease-free water were mixed to a total volume of 20 μl. The selected genes were amplified using the ABI 7300 Real-time PCR System (Applied Biosystems, Foster City, USA) and subsequently quantified by normalization to beta-actin and/or Rpl19. All primers used for quantitative real-time PCR are depicted in Table S3.

### Phagocytosis and Pinocytosis Assay

PECs were seeded in a 24 well plate (5 × 10^5^) and polarized for 72 h. In case of co-culture experiments, PECs were polarized for 24 h into M2-like macrophages and subsequently cultured for additional 24 h with OVA specific CD4^+^ Th1 cells. After washing three times with PBS, the cells were maintained in DMEM containing 10% FCS and either 1 mg/ml FITC-dextran (Sigma-Aldrich) or 1.25 × 10^6^/ml FluoSpheres™ Carboxylate-Modified Microspheres (2.0 μm; Thermo Fisher Scientific) to monitor pinocytosis and phagocytosis, respectively. Thereafter, PECs were harvested, stained with LIVE/DEAD® Fixable Yellow dye and analyzed by flow cytometry. Background values were determined upon co-incubation of cells with FITC labeled particles at 4°C and substracted from the values measured after co-culture at 37°C.

### IFN**γ** ELISA

Supernatants of co-cultured PECs were collected and the amount of secreted IFNγ was quantified using Mouse IFN gamma ELISA Ready-SET-Go! Kit (eBioscience, Waltham, USA) according to manufacturer's instructions.

### Peptides

Peptides (Table S4) were synthetized by Fmoc chemistry using a fully automated multiple synthesizer Syro II (MultiSynTech, Witten, Germany), followed by HPLC purification on a Kromasil 100–10C 10 μm 120A reverse phase column (20 × 150 mm). Eluted peptides were analyzed by HPLC and MS (Thermo Finnigan LCQ).

### Adoptive T Cell Transfer

Tumor cells were harvested, washed 3 times with PBS and adjusted to the desired titer. C57BL/6 Ly5.1 mice were injected s.c. into the right flank with 100 μl cell suspensions containing (2 × 10^5^) B16F10 IA^b−/−^ or OVA expressing B16F10 IA^b−/−^ cells, respectively. Tumor bearing C57BL/6 Ly5.1 mice received adoptive T cell transfer 8–10 days post tumor cell injection. Therefore, splenocytes of OT-II mice were resuspended in complete T cell medium and cultured in 24-Well plates (6 × 10^6^ cells per well in 2 ml) in the presence of 1 μg/ml IA^b^ restricted OVA peptide (aa 323–339) for 3 days. On day 2, 1 ml of culture supernatant was replenished and 1 day later cells were harvested, positively selected using CD4 (L3T4) MicroBeads (Miltenyi, Bergisch Gladbach, Germany) and adjusted to a 50 × 10^6^ cells/ml in PBS. One hundred microliter of this suspension was injected i.v. into the lateral tail vein of tumor bearing C57BL/6 Ly5.1 mice.

### Tumor Digestion and Isolation of Tumor Infiltrating Leukocytes

Tumors were harvested, transferred into hanks' balanced salt solution (HBSS, Sigma-Aldrich, St. Louis, USA) and cut into small pieces, followed by digestion with 0.5 mg/ml collagenase D (Hoffmann-La Roche, Basel, Switzerland), 10 μg/ml DNAse I (Sigma-Aldrich), 0.1 μg/ml TLCK (Sigma-Aldrich) and 10 mM HEPES buffer (Sigma-Aldrich) in HBSS for 1 h at 37°C on a shaker (200 rpm). Subsequently, tumor pieces were passed through a 70 μm cell strainer followed by passage through a 40 μm cell strainer and the single cell suspension was centrifuged at 300 × g for 10 min. The cell pellet was resuspended in 4 ml ACK lysing buffer (Thermo Fisher Scientific) followed by incubation for 2 min at RT. The erythrocyte lysis was stopped by adding 46 ml RPMI 1,640 Medium (Thermo Fisher Scientific). After another centrifugation step at 300 × g for 10 min, cells were resuspended in PBS, adjusted to the respective titer and transferred to 96-well U-bottom plates for subsequent immunofluorescence staining.

## Results

### Susceptibility of Polarized PECs to CD4^+^ Th1 Cell Recognition

**Figure 1:**
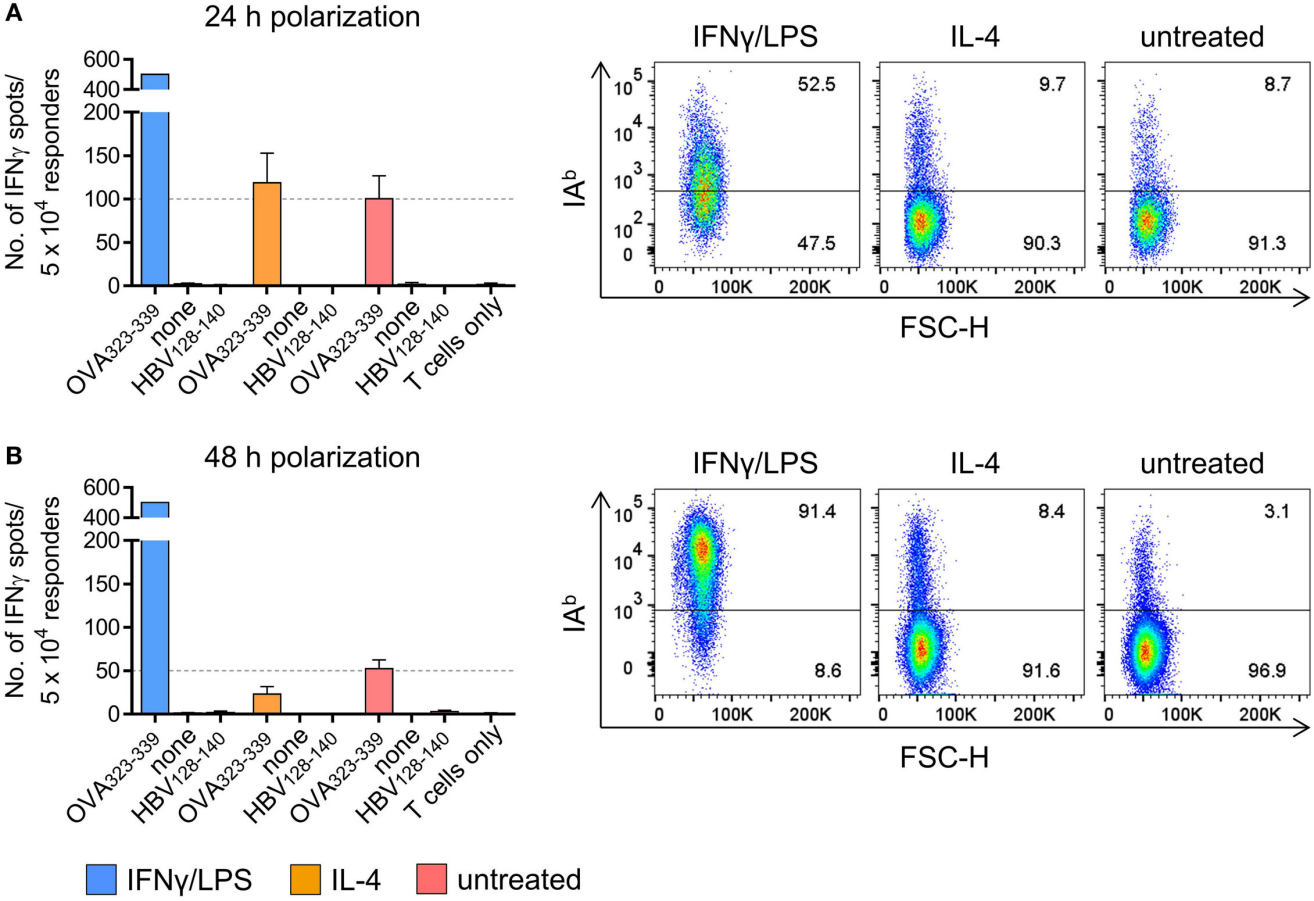
PECs polarized *in vitro* are susceptible to CD4^+^ T cell recognition. PECs were either left untreated or incubated with IFNγ/LPS or IL-4 to induce M1 or M2 polarization, respectively. Following incubation for 24 h **(A)** or 48 h **(B)**, PECs were pulsed with IA^b^ restricted epitope OVA_323−339_ or with HBV_128−140_ control epitope or were left without peptide (“none”). PECs were then co-cultured with an OVA-specific CD4^+^ T cell line for 24 h and T cell reactivity was analyzed by IFNγ ELISpot assay **(left)**. IA^b^ surface expression of PECs was determined by FACS **(right)**. Gating strategy: living cells → single cells (FSC-A vs. FSC-H) → F4/80^+^CD11b^+^ → IA^b^ vs. FSC-H.

In a set of pilot experiments, peritoneal exudate cells (PECs) were used as a surrogate for TAMs, thus minimizing the amount of tumor transplantation experiments required for TAM isolation. Flow cytometric analysis of cultured PECs revealed co-expression of the macrophage markers F4/80 and CD11b by more than 99 % of the cells (Figure S1). Treatment of PECs with LPS/IFNγ upregulated expression of M1-associated genes (Figure S2A) and induced IA^b^ surface expression (Figure S3), whereas IL-4 treatment resulted in M2-like polarization of PECs with enhanced M2-associated gene expression (Figure S2B) and unaltered IA^b^ surface expression (Figure S3). In order to analyze the susceptibility of polarized PECs to IA^b^ restricted recognition by CD4^+^ Th1 cells, PECs pretreated with LPS/IFNγ or IL4, respectively, were loaded with IA^b^ restricted OVA peptide and co-cultured over night with an OVA-specific CD4^+^ Th1 cell line established from peptide immunized C57BL/6 mice (Figure S4). As demonstrated by IFNγ ELISpot assays, peptide loaded PECs that had been polarized with LPS/IFNγ for 24 h were strongly recognized by OVA-specific CD4^+^ Th1 cells resulting in saturating spot numbers (i.e. >500 IFNγ spots) (Figure 1A, left). In contrast, IL-4 treated peptide pulsed PECs were significantly less susceptible to CD4^+^ T cell recognition, similarly to untreated PECs loaded with antigenic peptide (119 and 100 spots, respectively). Flow cytometric analysis revealed IA^b^ surface expression by 52.5% of PECs that had been polarized with LPS/IFNγ, whereas only 9.7% of IL-4 stimulated PECs and 8.7% of non-polarized PECs showed cell surface expression of IA^b^ molecules (Figure 1A, right). When polarized for 48 h, more than 90 % of LPS/IFNγ PECs had become IA^b^ positive showing IA^b^ expression levels that exceeded those of untreated PECs approximately 4-fold (MFI 10350 vs. MFI 2586) (Figure 1B, right). As already observed with short term polarized PECs (Figure 1A), saturating IFNγ spot formation was observed upon co-culture of CD4^+^ Th1 cells with peptide pulsed LPS/IFNγ treated macrophages.

### Cognate Interaction With CD4^+^ Th1 Cells Repolarizes M2-Like PECs

**Figure 2:**
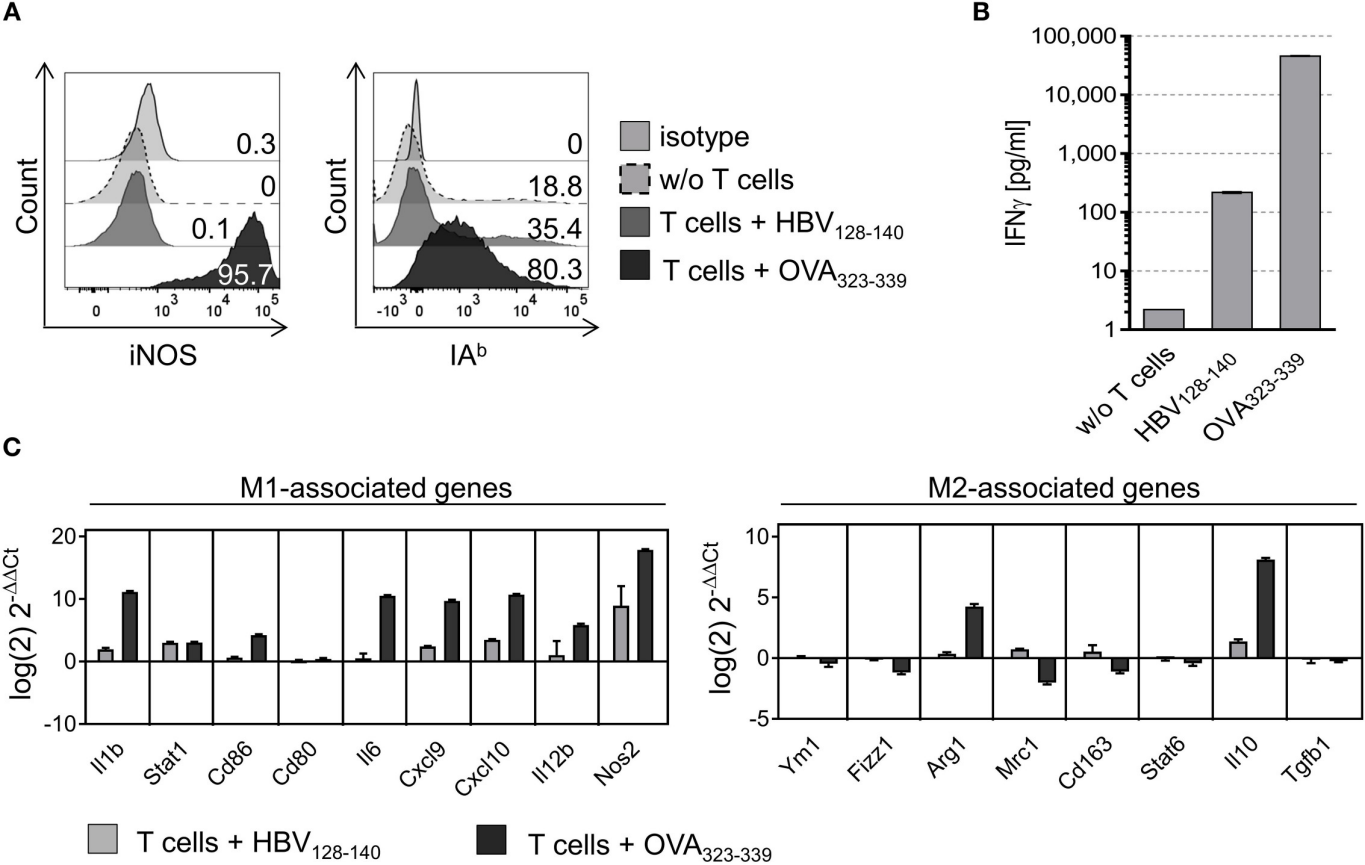
M2-like PECs are repolarized upon cognate interaction with CD4^+^ T cells. PECs were cultured with IL-4 for 24 h to induce M2 polarization. Subsequently, PECs were loaded with 1 μg/ml IA^b^ restricted epitope OVA_323−339_ or with HBV_128−140_ control epitope and co-cultured with an OVA-specific CD4^+^ T cell line for 24 h. **(A)** FACS analysis of co-cultured PECs using iNOS and IA^b^ specific monoclonal antibodies showing strong upregulation of both M1 markers. Gating strategy: living cells → single cells (FSC-A vs. FSC-H) → F4/80^+^CD11b^+^ → IA^b^ vs. FSC-H. **(B)** IFNγ ELISA showing that IFNγ concentration increased 20,000 fold in culture supernatants of M2 polarized PECs co-cultured with CD4^+^ T cells plus relevant peptide compared to PECs cultured without T cells. **(C)** Gene expression analysis revealed M1 repolarization of M2-like PECs upon co-culture with CD4^+^ T cells in presence of specific peptide. Expression data was normalized to PECs w/o T cell addition. Error bars represent SD of technical triplicates.

We next tested whether MHC II restricted T cell interaction would instruct PEC derived M2-like macrophages to acquire M1-like phenotype. Thus, PECs were treated with IL-4 for 24 h and polarization into M2-like macrophages was confirmed by flow cytometry and qPCR (see Figures S5A,B). M2-like PECs co-cultured with CD4^+^ Th1 cells in the presence of OVA peptide strongly upregulated both iNOS and IA^b^ expression, in contrast to M2-like PECs loaded with control peptide or to PECs cultured without T cells (Figure 2A). Interestingly, repolarization of M2-like PECs by cognate interaction with CD4^+^ Th1 cells, resulting in 95.7% iNOS positive and 80.3% IA^b^ positive PECs, was even more effective than polarization by external addition of IFNγ/LPS (compare Figure 2A and Figure S5A). Suspecting that IFNγ released by the CD4^+^ Th1 cells upon IA^b^ restricted interaction with M2-like PECs could be responsible for M1-repolarization, we determined IFNγ concentrations in culture supernatants by ELISA. As shown in Figure 2B, the IFNγ concentration was increased 210 fold in culture supernatants that included the OVA specific CD4^+^ T cell epitope compared to supernatants of co-cultures containing the irrelevant epitope (HBV_128−140_). Investigating the instructive effect of CD4^+^ Th1 recognition on gene expression level of M2-like PECs we found all M1-associated genes tested were upregulated after co-culture with CD4^+^ Th1 cells in presence of the OVA specific epitope, except *Cd80*, whereas most of the M2-associated genes were downregulated, with the exception of arginase 1 and IL-10 (Figure 2C). Notably, expression levels of arginase 1 and IL-10 were also enhanced in LPS/IFNγ treated PECs, but dropped in the case of IL-10, after prolonged time periods (48 h, see Figure S2B).

### M2-Like PECs Instructed by CD4^+^ Th1 Cells Gain M1-Like Function

**Figure 3:**
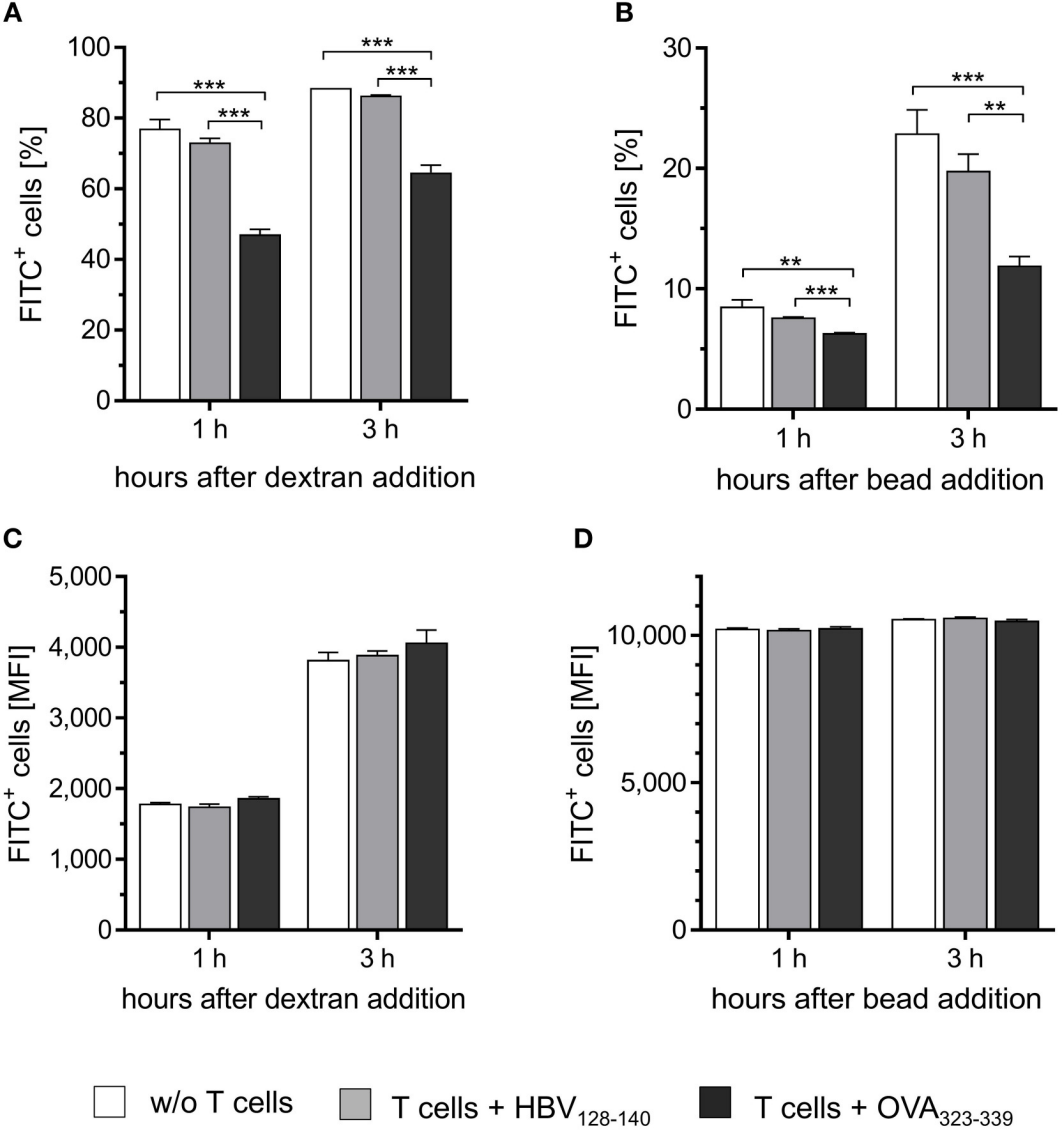
CD4^+^ T cells instruct M2-like PECs to acquire M1-like function. PECs were cultured with IL-4 for 24 h to induce M2 polarization. Subsequently, M2-like PECs were loaded with 1 μg/ml IA^b^ restricted OVA-specific peptide or with irrelevant control peptide and co-cultured with an OVA-specific CD4^+^ Th1 cell line for 24 h. Co-cultured PECs were then incubated with FITC-dextran **(A,C)** or with fluorescent latex beads **(B,D)** for different time periods (1 and 3 h) to monitor pinocytic and phagocytic capacity, respectively. Co-culture of M2 polarized PECs with CD4^+^ T h1 cells in the presence of OVA-specific peptide significantly decreased both, phagocytic as well as pinocytic activity compared to the control groups **(A,B)**, whereas median fluorescence intensities did not differ between the groups **(C,D)**. Significance was determined using One-way ANOVA with *post-hoc* Tukey test (95% CI, ^**^p < 0.01, ^***^p ≤ 0.001). Gating strategy: living cells → single cells (FSC-A vs. FSC-H) → FITC vs. FSC-H. Error bars represent SD of technical triplicates.

Next, we tested whether CD4^+^ Th1 cells would instruct M2-like PECs to acquire M1-like functionality. Thus, IL-4 treated PECs were loaded with IA^b^ restricted epitope OVA_323−339_ or with HBV_128−140_ control epitope and co-cultured with OVA-specific CD4^+^ Th1 cells. Subsequently, FITC-dextran was added to monitor pinocytic activity. As expected, the percentage of PECs taking up FITC-dextran was significantly reduced upon co-culture with CD4^+^ Th1 cells in presence of the relevant peptide, but remained unchanged if the control peptide was added (Figure 3A). On the other hand, the total amount of FITC-dextran taken up by the macrophages was equal among the different groups (Figure 3C), demonstrating that the cognate interaction with CD4^+^ T cells had reduced the frequency of pinocytic PECs (Figure 3A), without affecting their pinocytosis capacity.

Similar results were obtained after incubation of PECs with fluorescent latex beads. Already 1 h after incubation, the proportion of FITC positive cells was significantly reduced among the population of IL-4 treated PECs co-cultured with CD4^+^ T cells in the presence of relevant peptide compared to the PECs from the two control groups (Figure 3B). These effects became even more pronounced after incubation for 3 h. No differences in the total amount of phagocytosed beads were detected among the three groups of PECs (Figure 3D), similarly to the observations made when analyzing pinocytotic capacity (Figure 3C).

In summary, these gene expression analyses and functional assays clearly show that cognate interaction with CD4^+^ T cells instructs M2-like PECs to acquire M1-like phenotype and function *in vitro*.

### Adoptive Transfer of Tumor Antigen Specific CD4^+^ Th1 Cells Induces M1-Like Gene Expression in TAMs of IA^b^ Negative B16F10/OVA Tumors

**Figure 4:**
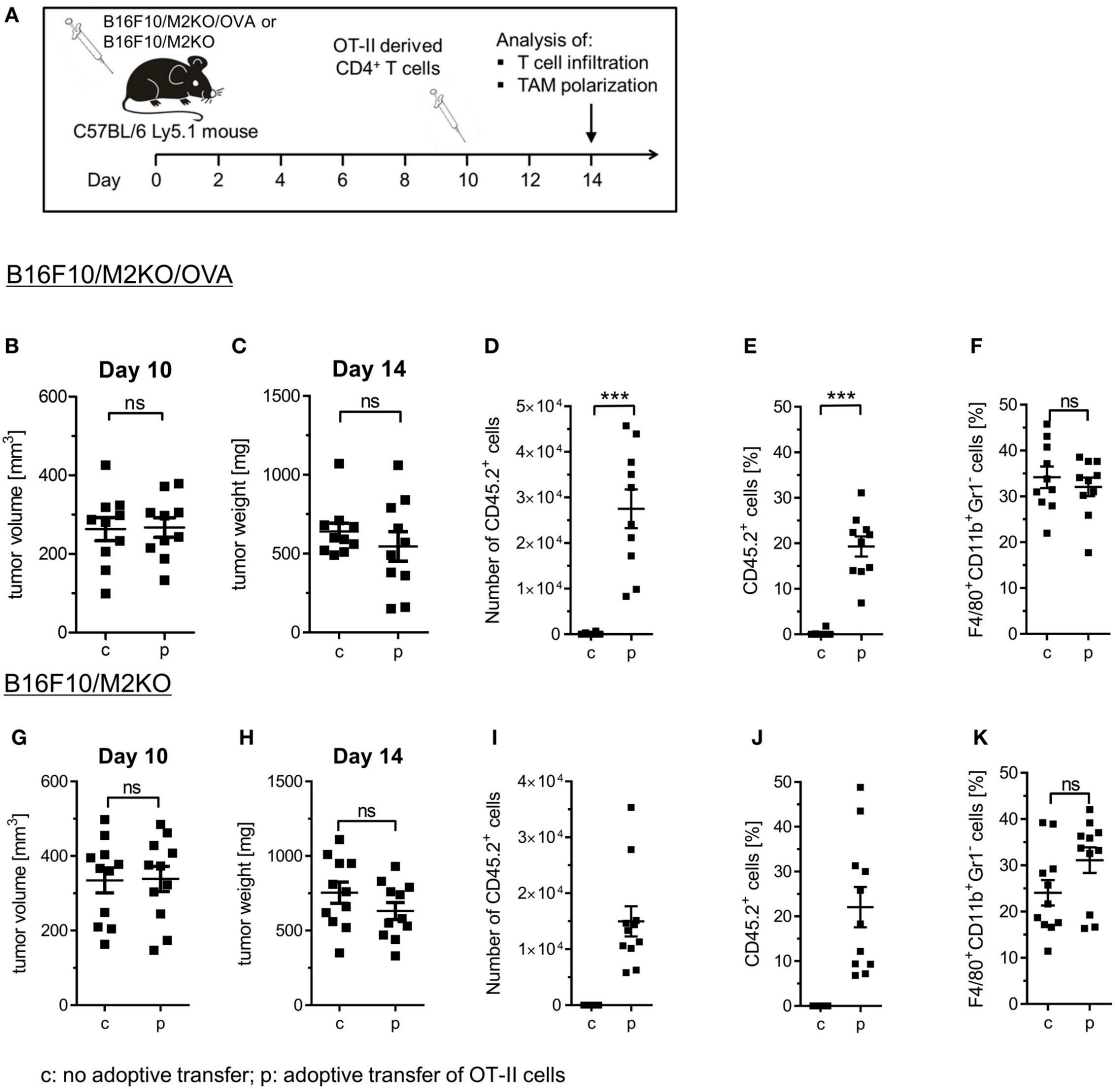
Immune cell composition of B16F10/M2KO/OVA and B16F10/M2KO tumors after adoptive transfer of OVA specific CD4^+^ T cells. **(A)** Experimental workflow. C57BL/6 Ly5.1 mice (*n* = 10–11) were injected s.c. with 2 × 10^5^ B16F10/M2KO/OVA cells **(B–F)** or B16F10/M2KO cells (**G–K**) respectively. Ten days post tumor inoculation, mice were injected i.v. with 5 × 10^6^ peptide activated OVA specific OT-II T cells (p), whereas control mice were left untreated (c). Mice were sacrificed on day 14 and tumors were analyzed by flow cytometry. Tumor volume **(B,G)** and tumor weight **(C,H)** determined 10 and 14 days, respectively, after tumor cell injection. The absolute numbers of infiltrating OT-II cells **(D, I)** as well as the proportion of adoptively transferred CD45.2^+^ OT-II cells among CD4^+^CD8^−^ TILs **(E,J)** and of F4/80^+^CD11b^+^Gr1^+^ TAMs among CD45^+^ cells **(F,K)** are shown. Statistical analysis was done by unpaired Student's *t*-test (95% CI, ns: not significant, ^***^p ≤ 0.001). Gating strategy: CD45^+^ → living cells → single cells (FSC-A vs. FSC-H) → F4/80^+^CD11b^+^Gr1^−^ or CD4^+^CD8^−^ → CD45.1 vs CD45.2.

**Figure 5:**
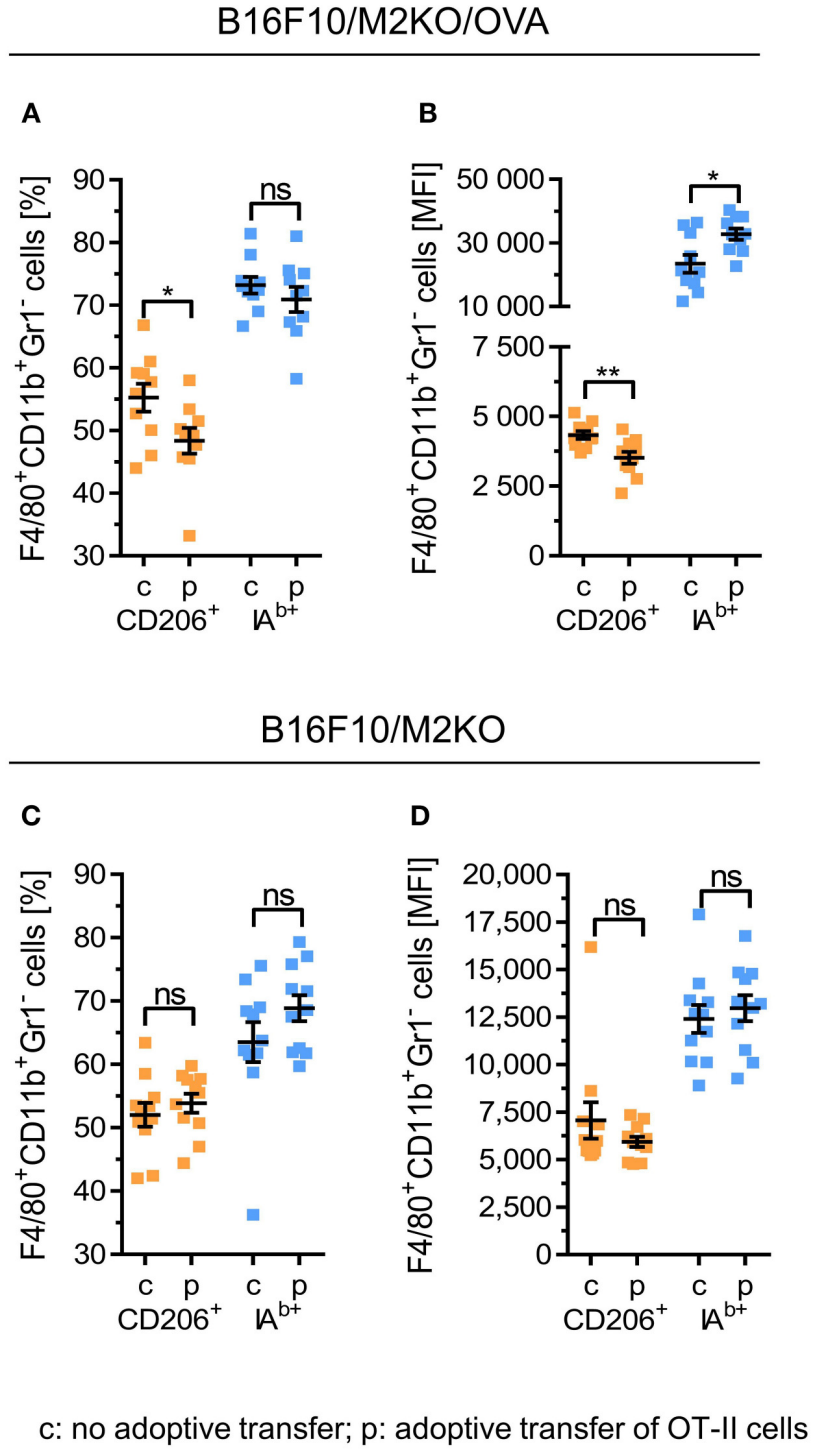
Adoptive transfer of OVA-specific CD4^+^ T cells repolarizes TAM phenotypes in B16F10/M2KO/OVA tumors. C57BL/6 Ly5.1 mice (*n* = 10–11) were injected s.c. either with 2 × 10^5^ B16F10/M2KO/OVA cells **(A,B)** or with OVA negative B16F10/M2KO cells **(C,D)**. After 10 days, mice bearing tumors of equal size were injected i.v. with 5 × 10^6^ peptide primed OT-II cells (p) or were left untreated (c), followed by flow cytometric analysis of tumor infiltrating immune cells on day 14. Frequencies of CD206^+^ and IA^b^ positive TAMs (F4/80^+^CD11b^+^Gr1^−^) **(A,C)** as well as cell surface expression levels of CD206^+^ and IA^b^ molecules **(B,D)** are depicted. Error bars represent SEM within each animal collective. Gating strategy: CD45^+^ → living cells → single cells (FSC-A vs. FSC-H) → F4/80^+^CD11b^+^Gr1^−^ → CD206 vs. IA^b^. Statistical analysis was done by unpaired Student's *t*-test (95% CI, ns: not significant, ^*^p ≤ 0.05, ^**^p < 0.01).

We then investigated whether OVA-specific CD4^+^ Th1 cells would promote differentiation of tumor associated macrophages also *in vivo*. Therefore, a murine melanoma model expressing OVA as tumor antigen was established upon transduction of a B16F10 derived IA^b^ KO clone M2KO (24) with an OVA encoding lentiviral construct. The resulting OVA expressing sub-clone A1 lacking IA^b^ surface expression (Figure S6), designated B16F10/M2KO/OVA throughout the paper (Figure S6C), was injected s.c. into C57BL/6 mice (Figure 4A). Ten days later, mice bearing tumors of equal size (Figures 4B,C,G,H) were randomized into two groups, one group receiving 5 × 10^6^ OT-II cells by i.v. injection and a control group that was left untreated. Analysis of TILs performed on explanted tumors 4 days post adoptive T cell transfer revealed that on average 2.7 × 10^4^ OT-II cells had reached the tumor (Figure 4D), representing 19.3% of the CD4^+^ TIL compartment in B16F10/M2KO/OVA tumors (Figure 4E). Interestingly, although the overall frequency of TAMs from control mice and treated animals showed no significant differences (Figure 4F), the frequency of CD206^+^ TAMs as well as the CD206 surface expression intensity were decreased (Figures 5A,B). At the same time, IA^b^ surface expression was significantly enhanced on TAMs from tumors of treated mice compared to TAMs from tumors of naïve mice (Figure 5B), although the overall frequency of IA^b^ positive TAMs appeared unaltered in both groups of mice (Figure 5A). Even though the frequencies of CD45.2^+^ CD4 T cells were similar between B16F10/M2KO and B16F10/M2KO/OVA tumors (see Figures 4E,J), the total number of infiltrating OT-II cells was significantly higher in OVA expressing tumors (Figures 4I,D). TAM frequency in B16F10/M2KO tumors of control and treated mice showed no significant differences (Figure 4K). Most importantly, adoptive transfer of OT-II cells did not alter the frequency of CD206^+^ TAMs nor their CD206 surface expression levels in OVA negative tumors (Figures 5C,D), suggesting that the degree of tumor infiltration by tumor antigen-specific CD4^+^ T cells as well as M1 polarization of TAMs might depend on cognate interaction with CD4^+^ T cells.

**Figure 6:**
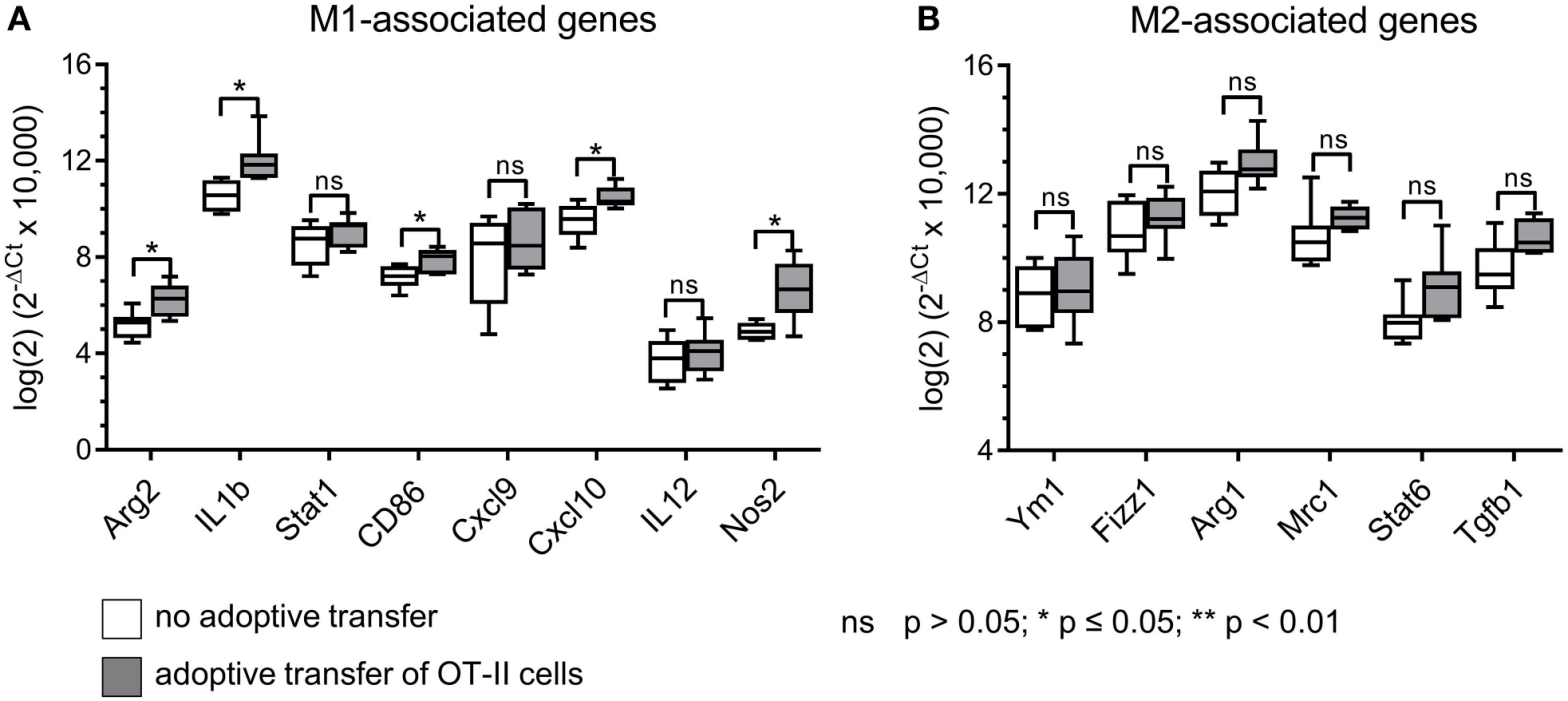
Adoptive transfer of OVA-specific CD4^+^ T cells induces M1-like gene expression in TAMs of B16F10/M2KO/OVA tumors. Gene expression analysis of sorted TAMs from B16F10/M2KO/OVA tumors of mice adoptively transferred with OT-II cells revealed significantly increased expression of M1-associated genes **(A)**, while expression of M2-associated genes showed no significant changes **(B)**. The box and whiskers plots extend from the smallest to the largest values and show the median, the 25th and the 75th percentiles. Significance was determined using unpaired two-tailed Student's *t*-test with Bonferroni-Holm *p-*value correction.

Having observed reduced frequencies of CD206^+^ TAMs with enhanced MHC II surface expression after adoptive OT-II cell transfer selectively in B16F10/M2KO/OVA tumors, we next performed gene expression analyses on TAMs freshly isolated from B16F10/M2KO/OVA tumors. As shown in Figure 6, transfer of OT-II cells resulted in TAMs with enhanced expression of the M1-associated genes *Arg2, Il1b, Cd86, Cxcl10*, and *Nos2* (Figure 6A), largely reflecting the M1-gene expression profile observed upon interaction with OVA-specific CD4^+^ T cells *in vitro* (Figure 2C). The expression pattern of M2-associated genes tested showed no significant changes upon adoptive transfer of OT-II cells (Figure 6B).

The results presented here thus show that transferred OT-II cells predominantly accumulate in OVA-expressing tumors, thereby increasing the frequencies of M1-like TAMs.

## Discussion

The functional role CD4^+^ T cells play in immunological tumor attack is complex (25). On the one hand, CD4^+^ T cells accumulating in tumors often belong to the Foxp3 expressing regulatory T cell subset, which can be induced by TAMs through IL-10 release as demonstrated in tumor tissues of ovarian cancer patients (26). Similarly, naïve T cells were shown to differentiate into IL-10 and TGFβ secreting Treg cells, when co-cultured with TAMs from glioblastoma patients (27). Conversely, CD4^+^ T cells can also gain tumor protective function as CD4^+^ T cells with cytotoxic capacity were shown to eliminate MHC II expressing tumor cells (28). Moreover, CD4^+^ T cells could mediate eradication of MHC II negative tumor cells through indirect recognition of tumor antigen or upon cooperation with NK cells (29, 30). Another tumor protective mode of action brought about by CD4^+^ T cells might be functional M1 polarization of TAMs through MHC II restricted interaction. Therefore, the aim of this study was to characterize CD4^+^ T cell mediated repolarization of M2-like macrophages *in vitro* and *in vivo*. While the instructive effect of CD4^+^ Th1 cells on M2-like macrophages could be directly shown *in vitro*, repolarization of TAMs by CD4^+^ Th1 cells *in vivo* was demonstrated indirectly through adoptive transfer of OT-II cells.

Repolarization of M2-like macrophages by CD4^+^ T cells has been shown before *in vitro* using human HPV specific T cell clones (31). Our data not only confirm these results, but furthermore show that cognate CD4^+^ Th1 cell/M2 interaction has also consequences on functional level, since both pino- and phagocytosis activities of instructed PECs were reduced. Our gene expression analyses performed on PECs repolarized by OVA-specific CD4^+^ Th1 cells revealed enhanced expression levels of M1-associated genes (e.g., *Il1b, Cd86, Il6. Cxcl9, Cxcl10, Il12b, Nos2*) and reduced levels of M2-associated genes (e.g., *Fizz1, Mrc1, Cd163*; Figure 2). Unexpectedly, expression of Arg-1 and IL-10 was enhanced, both representing classical M2-associated genes. In fact, Arginase 1 expression has been reported in chronically infected M1-like rat peritoneal macrophages (32) and in murine PECs stimulated with LPS (33). It was suggested that Arg-1 expression in M1-like macrophages might represent a possible safety mechanism that restricts the amount of arginine accessible for iNOS mediated oxidation, thereby avoiding generation of excessive NO concentrations that might become harmful to the host tissue (34). Expression of IL-10 is a characteristic of M2-like macrophages, yet IL-10 expression has been also described in so called regulatory macrophages (35) and in M2b macrophages known to express both immune suppressive as well as inflammatory cytokines (36). However, induction of M2b like macrophages appears unlikely in our setting, since this process depends on IFNγ in conjunction with Fc receptor binding of immune complexes (36), which were lacking in our co-culture experiments with CD4^+^ Th1 cells.

Repolarization of M2-like TAMs rather than depletion of the entire TAM population might represent a physiological way to neutralize the immune-suppressive tumor micro milieu (37), thus facilitating CTL mediated tumor attack. This was impressively demonstrated in a study by Klug et al. showing that M1-like macrophages can hold significant immuno-stimulatory potential rendering them indispensable for the therapeutic success of low dose irradiation in a murine pancreatic cancer model (16). Macrophage repolarization induced with cytokines or chemokines has been extensively described *in vitro* and a substantial amount of data on gene and protein expression patterns as well as functional properties have been published (22, 23). M1 polarization of TAMs *in vivo* upon interaction with tumor antigen specific CD4^+^ T cells has also been demonstrated in a murine multiple myeloma model based on TCR transgenic SCID mice. Such interaction mediated rejection of MHC II negative myelomas (19, 38), thereby demonstrating tumor cell killing by inflammatory macrophages as a consequence of CD4^+^ Th1 cell mediated TAM instruction (39). In our study, we used fully immune competent C57BL/6 mice, as we were interested to investigate the effect of a CD4^+^ Th1 / TAM interaction on TAM differentiation in the context of a native immune system. Also, we avoided Matrigel during tumor cell transplantation to rule out possible side effects potentially caused by traces of chemokines present within the Matrigel preparation such as TGF-β and VEGF (38).

Our *in vivo* experiments show that adoptively transferred OT-II cells selectively accumulating within OVA-expressing tumors enhanced the frequencies of TAMs with M1-associated gene and surface marker expression. Although we assume that this shift in the TAM profile resulted from cognate interaction between OT-II cells and M2-like TAMs *in vivo*, interactions with other MHC II expressing cells resulting in IFNγ release from stimulated OT-II cells appear conceivable as well. In fact, the function of dendritic cells (DCs) in uptake and processing of exogenous tumor antigen for MHC II restricted epitope presentation is well established (40, 41).

Myeloid derived suppressor cells (MDSCs) are generally considered to express only low levels of MHC II molecules on their surface (42, 43). Still, MDSCs were shown to interact with CD4^+^ T cells inducing their differentiation into regulatory T cells (44). Recently, it was shown that MDSC from autophagy deficient mice bearing B16OVA tumors effectively stimulated adoptively transferred OT-II cells, demonstrating that MDSC can take up and process exogenous tumor antigen for MHC II restricted epitope presentation (45). However, this effect was less pronounced among MDSC from wildtype mice, and MHC II surface expression can substantially differ between MDSCs in the peripheral lymphatic organs and those infiltrating the tumor (46). It thus remains speculative whether and to which extent a potential MDSC/OT II cell interaction is involved to the polarizing effect observed *in vivo* following OT-II cell transfer.

Much less is known about the function of B cells as MHC II expressing antigen presenting cells (APCs) in the tumor microenvironment. In fact, processing of exogenous antigens for MHC II restricted epitope presentation after B cell receptor mediated uptake or upon micropinocytosis has been described (47, 48). However, since we did not analyze the B cell compartment in our study, it remains open whether interactions between B cells and transferred OT II cells might have affected TAM polarization in B16F10/M2KO/OVA tumors.

When analyzing the effect of adoptively transferred OT-II cells on the cellular composition and differentiation stage of TAMs in B16F10/M2KO/OVA tumors, a gene expression pattern was observed among freshly isolated TAMs that was reminiscent of the M1 associated gene expression profile determined *in vitro* upon co-culture of peptide loaded M2-like PECs with OVA-specific CD4^+^ T cells, namely enhanced expression of *Arg-2, Il-1b, Cd86, Nos-2b* as well as increased IA^b^ surface expression and intracellular iNOS expression. We thus assume that the differentiation of TAMs selectively observed in OVA expressing B16F10/M2KO/OVA tumors was caused by IFNγ released from CD4^+^ Th1 cells as a consequence of tumor antigen-specific TAM / CD4^+^ Th1 interaction *in vivo*. This hypothesis is also supported by a recent study performed on human TAMs in premalignant oral cancer lesions in which immuno-histochemical analyses lead to the conclusion that tumor infiltrating Th1 cells mediated polarization of CD163^+^ TAMs toward M1 through IFN secretion (49).

**Figure 7:**
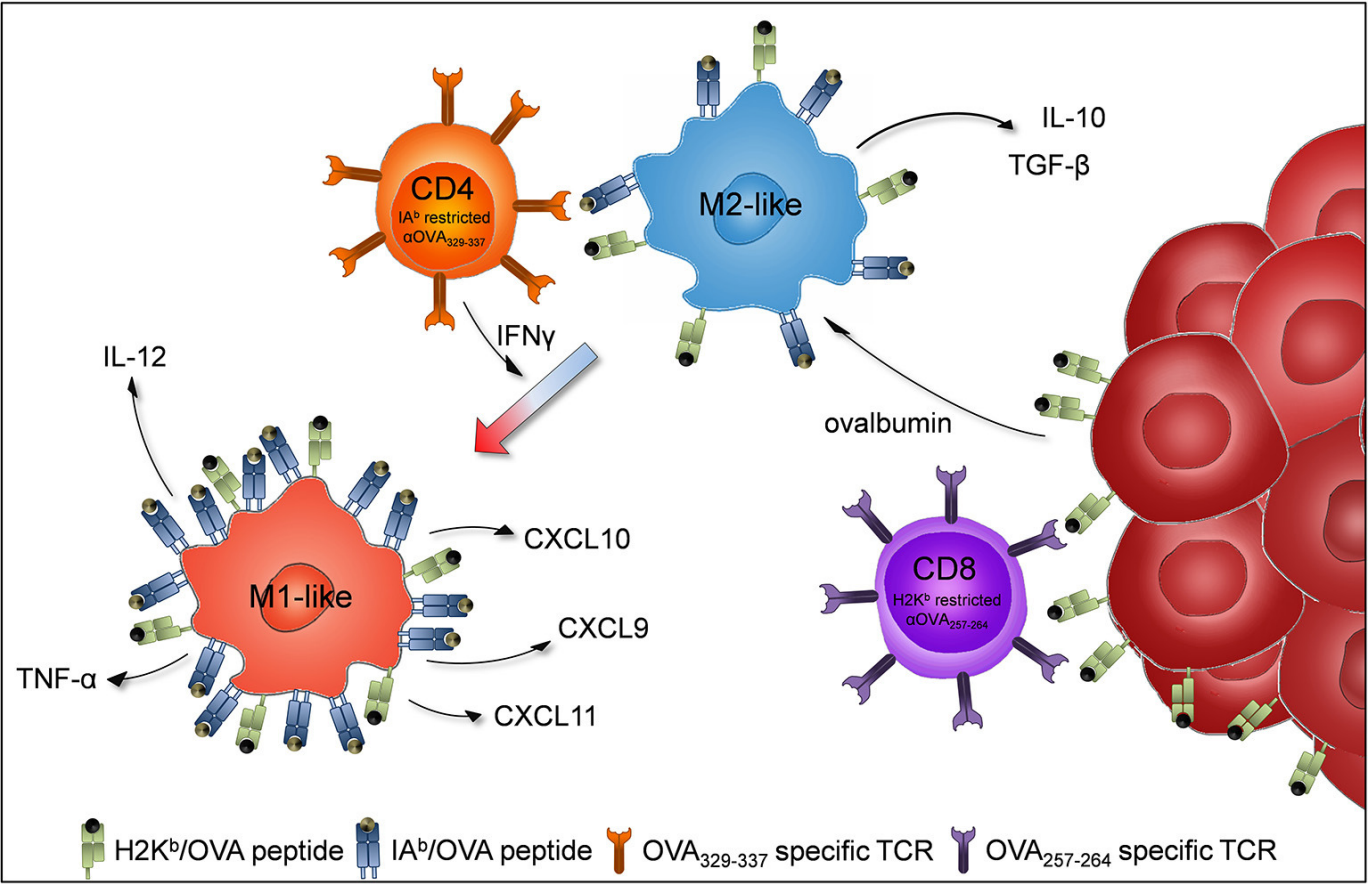
Proposed model of TAM repolarization upon adoptive transfer of tumor antigen-specific CD4^+^Th1 cells. B16F10 cells (dark red) are injected subcutaneously into C57BL/6J mice to induce tumor growth. Tumor infiltrating M2-like macrophages (blue) engulf ovalbumin released by tumor cells and present MHC class II (IA^b^) restricted OVA epitopes to adoptively transferred OVA specific CD4^+^ Th1 cells (orange). The activated CD4^+^ Th1 cells secrete IFNγ which induces macrophages to polarize to proinflammatory M1-like macrophages (light red).

In summary, our results show that the adoptive transfer of OT-II cells increased the proportion of M1-like macrophages within transplanted B16F10/M2KO/OVA tumors. Thus, cognate interaction between tumor antigen specific CD4^+^ Th1 cells and TAMs might promote recreation of an immuno-stimulatory tumor micro milieu, thereby facilitating efficient anti-tumoral immune attack (Figure 7).

## Ethics Statement

This study was carried out in accordance with the recommendations of German animal protection law. The protocol was approved by the inhouse animal well-fare officer and by District Government in Karlsruhe, Germany (approval ID 35–9158.81/G-211/16).

## Author Contributions

DE, KD, WO, and SE designed the experiments and performed data analyses. DE and ED performed the experiments. DE, KD, and RK contributed analysis tools. DE, WO, and SE wrote the paper.

### Conflict of Interest Statement

DE is currently affiliated with BioNTech however this study was conducted and completed whilst affiliated with the German Cancer Research Center. The remaining authors declare that the research was conducted in the absence of any commercial or financial relationships that could be construed as a potential conflict of interest.

## References

[1] Dunn GP Old LJ Schreiber RD. The immunobiology of cancer immunosurveillance and immunoediting. Immunity (2004) 21:137–48. 10.1016/j.immuni.2004.07.017 15308095

[2] Morvan MG Lanier LL. NK cells and cancer: you can teach innate cells new tricks. Nat Rev Cancer (2015) 16:7. 10.1038/nrc.2015.5 26694935

[3] Long KB Beatty GL. Harnessing the antitumor potential of macrophages for cancer immunotherapy. OncoImmunology (2013) 2:e26860. 10.4161/onci.26860 24498559 PMC3902119

[4] Xie Y Akpinarli A Maris C Hipkiss EL Lane M Kwon EK. Naive tumor-specific CD4(+) T cells differentiated in vivo eradicate established melanoma. J Exp Med. (2010) 207:651–67. 10.1084/jem.20091921 20156973 PMC2839147

[5] Quezada SA Simpson TR Peggs KS Merghoub T Vider J Fan X. Tumor-reactive CD4+ T cells develop cytotoxic activity and eradicate large established melanoma after transfer into lymphopenic hosts. J Exp Med. (2010) 207:637–50. 10.1084/jem.20091918 20156971 PMC2839156

[6] Ugel S De Sanctis F Mandruzzato S Bronte V. Tumor-induced myeloid deviation: when myeloid-derived suppressor cells meet tumor-associated macrophages. J Clin Invest. (2015) 125:3365–76. 10.1172/JCI80006 26325033 PMC4588310

[7] Cassetta L Kitamura T. Targeting tumor-associated macrophages as a potential strategy to enhance the response to immune checkpoint inhibitors. Front Cell Dev Biol. (2018) 6:38. 10.3389/fcell.2018.00038 29670880 PMC5893801

[8] Biswas SK Mantovani A. Macrophage plasticity and interaction with lymphocyte subsets: cancer as a paradigm. Nat Immunol. (2010) 11:889–96. 10.1038/ni.1937 20856220

[9] Vesely MD Kershaw MH Schreiber RD Smyth MJ. Natural innate and adaptive immunity to cancer. Annu Rev Immunol. (2011) 29:235–71. 10.1146/annurev-immunol-031210-101324 21219185

[10] Lewis C Pollard J. Distinct role of macrophages in different tumor microenvironments. Cancer Res. (2006) 66:605–12. 10.1158/0008-5472.CAN-05-4005 16423985

[11] Mantovani A Marchesi F Malesci A Laghi L Allavena P. Tumour-associated macrophages as treatment targets in oncology. Nat Rev Clin Oncol. (2017) 14:399–416. 10.1038/nrclinonc.2016.217 28117416 PMC5480600

[12] Mantovani A Allavena P. The interaction of anticancer therapies with tumor-associated macrophages. J Exp Med. (2015) 212:435–45. 10.1084/jem.20150295 25753580 PMC4387285

[13] Pollard J. Tumour-educated macrophages promote tumour progression and metastasis. Nat Rev Cancer (2004) 4:71–8. 10.1038/nrc1256 14708027

[14] Zhang Q-w Liu L Gong C-y Shi H-s Zeng Y-h Wang X-z. Prognostic significance of tumor-associated macrophages in solid tumor: a meta-analysis of the literature. PLoS ONE (2012) 7:e50946. 10.1371/journal.pone.0050946 23284651 PMC3532403

[15] Tang X. Tumor-associated macrophages as potential diagnostic and prognostic biomarkers in breast cancer. Cancer Lett. (2013) 332:3–10. 10.1016/j.canlet.2013.01.024 23348699

[16] Klug F Prakash H Huber Peter E Seibel T Bender N Halama N. Low-Dose irradiation programs macrophage differentiation to an iNOS+/M1 phenotype that orchestrates effective T cell immunotherapy. Cancer Cell (2013) 24:589–602. 10.1016/j.ccr.2013.09.014 24209604

[17] De Palma M Coukos G Hanahan D. A new twist on radiation oncology: low-dose irradiation elicits immunostimulatory macrophages that unlock barriers to tumor immunotherapy. Cancer Cell (2013) 24:559–61. 10.1016/j.ccr.2013.10.019. 24229704

[18] Heusinkveld M de Vos van Steenwijk PJ Goedemans R Ramwadhdoebe TH Gorter A Welters MJP. M2 Macrophages induced by prostaglandin E2 and IL-6 from cervical carcinoma are switched to activated M1 macrophages by CD4+ Th1 cells. J Immunol. (2011) 187:1157–65. 10.4049/jimmunol.1100889 21709158

[19] Corthay A Skovseth DK Lundin KU Rosjo E Omholt H Hofgaard PO. Primary antitumor immune response mediated by CD4+ T cells. Immunity (2005) 22:371–83. 10.1016/j.immuni.2005.02.003 15780993

[20] Tveita AA Schjesvold FH Sundnes O Haabeth OAW Haraldsen G Bogen B. Indirect CD4+ T-cell-mediated elimination of MHC IINEG tumor cells is spatially restricted and fails to prevent escape of antigen-negative cells. Eur J Immunol. (2014) 44:2625–37. 10.1002/eji.201444659 24846412

[21] Veremeyko T Yung AWY Anthony DC Strekalova T Ponomarev ED Early growth response gene-2 is essential for M1 and M2 macrophage activation and plasticity by modulation of the transcription factor CEBPβ. Front Immunol. (2018) 9:2515 10.3389/fimmu.2018.02515 30443252 PMC6221966

[22] Derlindati E Dei Cas A Montanini B Spigoni V Curella V Aldigeri R. Transcriptomic analysis of human polarized macrophages: more than one role of alternative activation? PLoS ONE (2015) 10:e0119751. 10.1371/journal.pone.0119751 25799240 PMC4370704

[23] Tarique AA Logan J Thomas E Holt PG Sly PD Fantino E. Phenotypic, functional, and plasticity features of classical and alternatively activated human macrophages. Am J Res Cell Mol Biol. (2015) 53:676–88. 10.1165/rcmb.2015-0012OC 25870903

[24] Das K Eisel D Lenkl C Goyal A Diederichs S Dickes E. Generation of murine tumor cell lines deficient in MHC molecule surface expression using the CRISPR/Cas9 system. PLoS ONE (2017) 12:e0174077. 10.1371/journal.pone.0174077 28301575 PMC5354463

[25] Ahrends T Borst J The opposing roles of CD4+ T cells in anti-tumour immunity. Immunology (2018) 154:582–92. 10.1111/imm.12941 PMC6050207 29700809

[26] Zhu Q Wu X Wu Y Wang X. Interaction between Treg cells and tumor-associated macrophages in the tumor microenvironment of epithelial ovarian cancer. Oncol Rep. (2016) 36:3472–8. 10.3892/or.2016.5136 27748885

[27] Li Z Liu X Guo R Wang P. CD4+Foxp3– type 1 regulatory T cells in glioblastoma multiforme suppress T cell responses through multiple pathways and are regulated by tumor-associated macrophages. Int J Biochem Cell Biol. (2016) 81:1–9. 10.1016/j.biocel.2016.09.013 27644153

[28] Takeuchi A Saito T. CD4 CTL, a cytotoxic subset of CD4+ T cells, their differentiation and function. Front Immunol. (2017) 8:194. 10.3389/fimmu.2017.00194 28280496 PMC5321676

[29] Shklovskaya E Terry AM Guy TV Buckley A Bolton HA Zhu E. Tumour-specific CD4 T cells eradicate melanoma via indirect recognition of tumour-derived antigen. Immunol Cell Biol. (2016) 94:593–603. 10.1038/icb.2016.14 26837456

[30] Perez-Diez A Joncker NT Choi K Chan WF Anderson CC Lantz O. CD4 cells can be more efficient at tumor rejection than CD8 cells. Blood (2007) 109:5346–54. 10.1182/blood-2006-10-051318 17327412 PMC1890845

[31] Heusinkveld M van der Burg S. Identification and manipulation of tumor associated macrophages in human cancers. J Trans Med. (2011) 9:216. 10.1186/1479-5876-9-216 22176642 PMC3286485

[32] Sonoki T Nagasaki A Gotoh T Takiguchi M Takeya M Matsuzaki H. Coinduction of nitric-oxide synthase and arginase I in cultured rat peritoneal macrophages and rat tissues in vivo by lipopolysaccharide. J Biol Chem. (1997) 272:3689–93. 9013624 10.1074/jbc.272.6.3689

[33] Ryan JL Yohe WB Morrison DC. Stimulation of peritoneal cell arginase by bacterial lipopolysaccharides. Am J Pathol. (1980) 99:451–61. 6990772 PMC1903489

[34] Modolell M Corraliza IM Link F Soler G Eichmann K Reciprocal regulation of the nitric oxide synthase/arginase balance in mouse bone marrow-derived macrophages by TH 1 and TH 2 cytokines. Eur J Immunol. (1995) 25:1101–4. 10.1002/eji.1830250436 7537672

[35] Suzuki H Hisamatsu T Chiba S Mori K Kitazume MT Shimamura K. Glycolytic pathway affects differentiation of human monocytes to regulatory macrophages. Immunol Lett. (2016) 176:18–27. 10.1016/j.imlet.2016.05.009 27208804

[36] Martinez FO Sica A Mantovani A Locati M. Macrophage activation and polarization. Front Biosci. (2008) 13:453–61. 10.2741/2692 17981560

[37] Zheng X Turkowski K Mora J Brune B Seeger W Weigert A. Redirecting tumor-associated macrophages to become tumoricidal effectors as a novel strategy for cancer therapy. Oncotarget (2017) 8:48436–52. 10.18632/oncotarget.17061 28467800 PMC5564660

[38] Haabeth OA Lorvik KB Hammarstrom C Donaldson IM Haraldsen G Bogen B. Inflammation driven by tumour-specific Th1 cells protects against B-cell cancer. Nat Commun. (2011) 2:240. 10.1038/ncomms1239 21407206 PMC3072106

[39] Tveita A Fauskanger M Bogen B Haabeth OA. Tumor-specific CD4+ T cells eradicate myeloma cells genetically deficient in MHC class II display. Oncotarget (2016) 7:67175–82. 10.18632/oncotarget.11946 27626487 PMC5341866

[40] Veglia F Gabrilovich DI. Dendritic cells in cancer: the role revisited. Curr Opin Immunol. (2017) 45:43–51. 10.1016/j.coi.2017.01.002 28192720 PMC5449252

[41] Nouri-Shirazi M Banchereau J Bell D Burkeholder S Kraus ET Davoust J. Dendritic cells capture killed tumor cells and present their antigens to elicit tumor-specific immune responses. J Immunol. (2000) 165:3797–803. 10.4049/jimmunol.165.7.3797 11034385

[42] Poschke I Mougiakakos D Hansson J Masucci GV Kiessling R. Immature immunosuppressive CD14^+^HLA-DR^−/*low*^ Cells in Melanoma Patients Are Stat3^hi^ and Overexpress CD80, CD83, and DC-sign. Cancer Research (2010) 70:4335–45. 10.1158/0008-5472.can-09-3767 20484028

[43] Filipazzi P Valenti R Huber V Pilla L Canese P Iero M. Identification of a new subset of myeloid suppressor cells in peripheral blood of melanoma patients with modulation by a granulocyte-macrophage colony-stimulation factor-based antitumor vaccine. J Clin Oncol. (2007) 25:2546–53. 10.1200/JCO.2006.08.5829 17577033

[44] Serafini P Mgebroff S Noonan K Borrello I. Myeloid-derived suppressor cells promote cross-tolerance in B-cell lymphoma by expanding regulatory T cells. Cancer Res. (2008) 68:5439–49. 10.1158/0008-5472.CAN-07-6621 18593947 PMC2887390

[45] Alissafi T Hatzioannou A Mintzas K Barouni RM Banos A Sormendi S. Autophagy orchestrates the regulatory program of tumor-associated myeloid-derived suppressor cells. J Clin Inves. (2018) 128:3840–52. 10.1172/JCI120888 29920188 PMC6118632

[46] Nagaraj S Nelson A Youn J-i Cheng P Quiceno D Gabrilovich DI. Antigen-specific CD4+ T cells regulate function of myeloid-derived suppressor cells in cancer via retrograde MHC class II signaling. Cancer Res. (2012) 72:928–38. 10.1158/0008-5472.CAN-11-2863 22237629 PMC4062074

[47] Nashar TO Drake JR. The pathway of antigen uptake and processing dictates MHC class II-mediated B cell survival and activation. J Immunol. (2005) 174:1306–16. 10.4049/jimmunol.174.3.1306 15661887

[48] Becker HJ Kondo E Shimabukuro-Vornhagen A Theurich S Bergwelt-Baildon MS. Processing and MHC class II presentation of exogenous soluble antigen involving a proteasome-dependent cytosolic pathway in CD40-activated B cells. Eur J Haematol. (2016) 97:166–74. 10.1111/ejh.12699 26561366

[49] Mori K Haraguchi S Hiori M Shimada J Ohmori Y. Tumor-associated macrophages in oral premalignant lesions coexpress CD163 and STAT1 in a Th1-dominated microenvironment. BMC Cancer (2015) 15:573. 10.1186/s12885-015-1587-0 26242181 PMC4525742

