# Supplementary material for: Cognate Interaction With CD4+ T Cells Instructs Tumor-Associated Macrophages to Acquire M1-Like Phenotype
Source: Front Immunol. 2019 Feb 22;10:219. doi: 10.3389/fimmu.2019.00219 (PMC6395406; doi:10.3389/fimmu.2019.00219)
Supplement: Supplementary file 1 [file Data_Sheet_1.pdf]

Supplemental Material to the article

Cognate interaction with CD4$^+$ T cells instructs tumor-associated macrophages to acquire M1-like phenotype

David Eisel[1,2,3], Krishna Das[1,2,4], Elke Dickes[1], Rainer König[5], Wolfram Osen[1], Stefan B. Eichmüller[1]

[1] GMP & T Cell Therapy Unit, German Cancer Research Center (DKFZ), Heidelberg, Germany

[2] Biosciences Faculty, University of Heidelberg, Germany

[3] Biopharmaceutical New Technologies (BioNTech) Corporation, Mainz, Germany

[4] Division of Virology, Innsbruck Medical University, Innsbruck, Austria

[5] Integrated Research and Treatment Center for Sepsis Control and Care (CSCC), Jena University Hospital, Jena, Germany

List of abbreviations

APC	antigen presenting cell
B16F10	murine melanoma cell line
B16F10/M2KO	IA^b knock-out clone derived from B16F10
B16F10/M2KO/Ova	Ovalbumin expressing clone derived from B16F10/M2KO
CTL	cytotoxic T lymphocyte
CXCL	C-X-C motif chemokine
DCs	dendritic cells
FoxP3	forkhead box P3
IFNγ	interferon-gamma
iNOS	inducible nitric oxide synthase
M2KO	MHC II knockout
MDSC	myeloid-derived suppressor cells
OVA	Ovalbumin
OT-II	CD4+ T cells from TCR transgenic mouse strain recognizing IA^b restricted OVA peptide (323-339)
PECs	peritoneal exudate cells
PRR	pattern recognition receptors
SCID	severe combined immunodeficiency
TAM	tumor associated macrophages
TCR	T cell receptor
TGF	transforming growth factor
TIL	tumor infiltrating lymphocyte
TLCK	N-alpha-tosyl-L-lysinyl-chloromethylketone
TME	tumor microenvironment
Treg	regulatory T cell
VEGF	vascular endothelial growth factor

Supplemental Figures

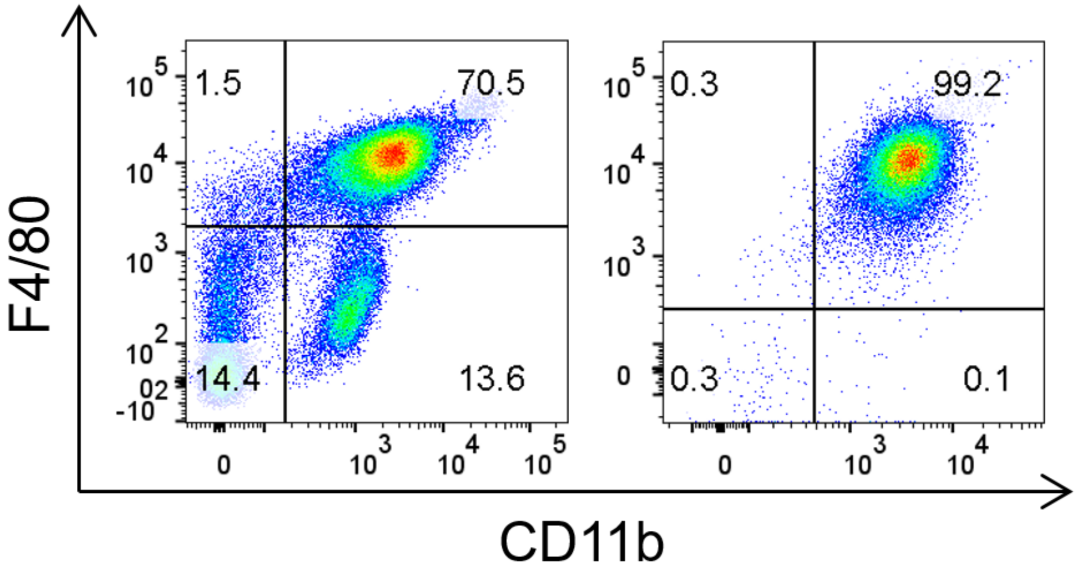

Figure S1. PECs contain a high proportion of macrophages. PECs of C57BL/6 mice injected i.p. with 1 ml of a 3 % thioglycollate solution four days before, were isolated and stained with monoclonal antibodies specific for F4/80 and CD11b resulting in 70.5 % F4/80$^+$CD11b$^+$ macrophages (left) as determined by flow cytometry. Upon short term culture for 2 h and subsequent medium exchange more than 99 % of all PECs co-expressed F4/80$^+$CD11b$^+$ (right). Gating strategy was as follows: living cells → single cells (FSC-A vs. FSC-H) → F4/80 vs. CD11b.

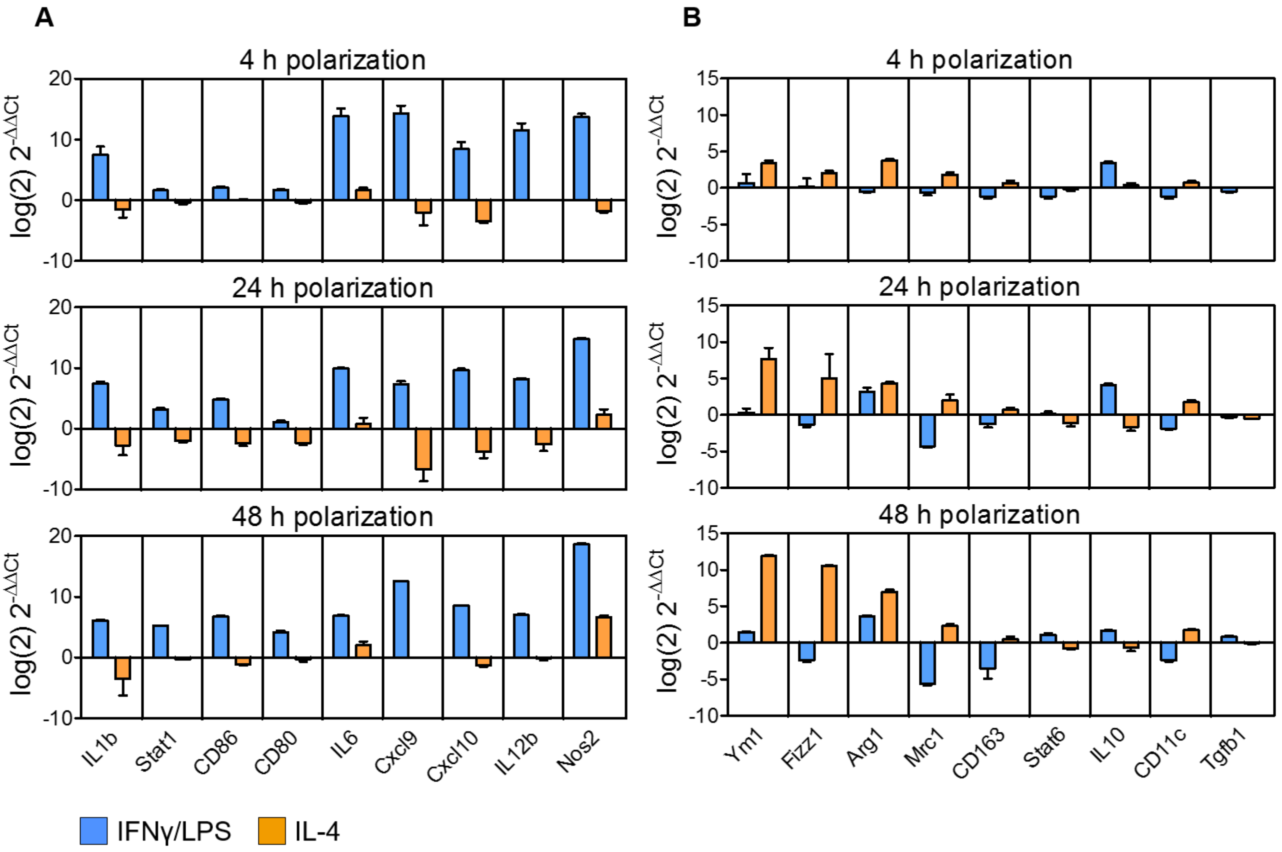

Figure S2. Gene expression analysis confirms M1/M2 phenotype of PECs polarized *in vitro.* PECs were polarized upon stimulation with LPS/IFNγ or IL-4, respectively for 4 h, 24 h and 48 h. RNA was isolated at each time point and gene expression was measured by quantitative real-time PCR. Gene expression was first normalized to beta-actin expression ($2^{-\Delta Ct}$) and subsequently normalized to expression data obtained from untreated PECs ($2^{-\Delta\Delta Ct}$). (**A**) M1-associated genes; (**B**) M2-associated genes. Error bars represent SD of technical triplicates.

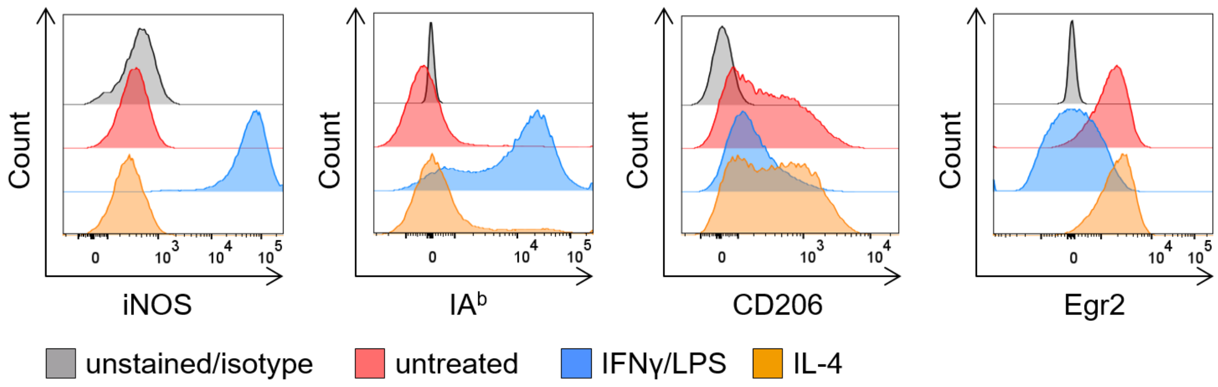

Figure S3. Surface marker expression profiles demonstrate polarization of PECs. PECs were polarized by treatment with LPS/IFNγ or IL-4, respectively for 48 h. Expression of iNOS, IA^b, CD206 and Egr2 was determined by flow cytometry. Gating strategy: living cells → single cells (FSC-A vs. FSC-H) → F4/80$^+$CD11b$^+$ → marker vs. count.

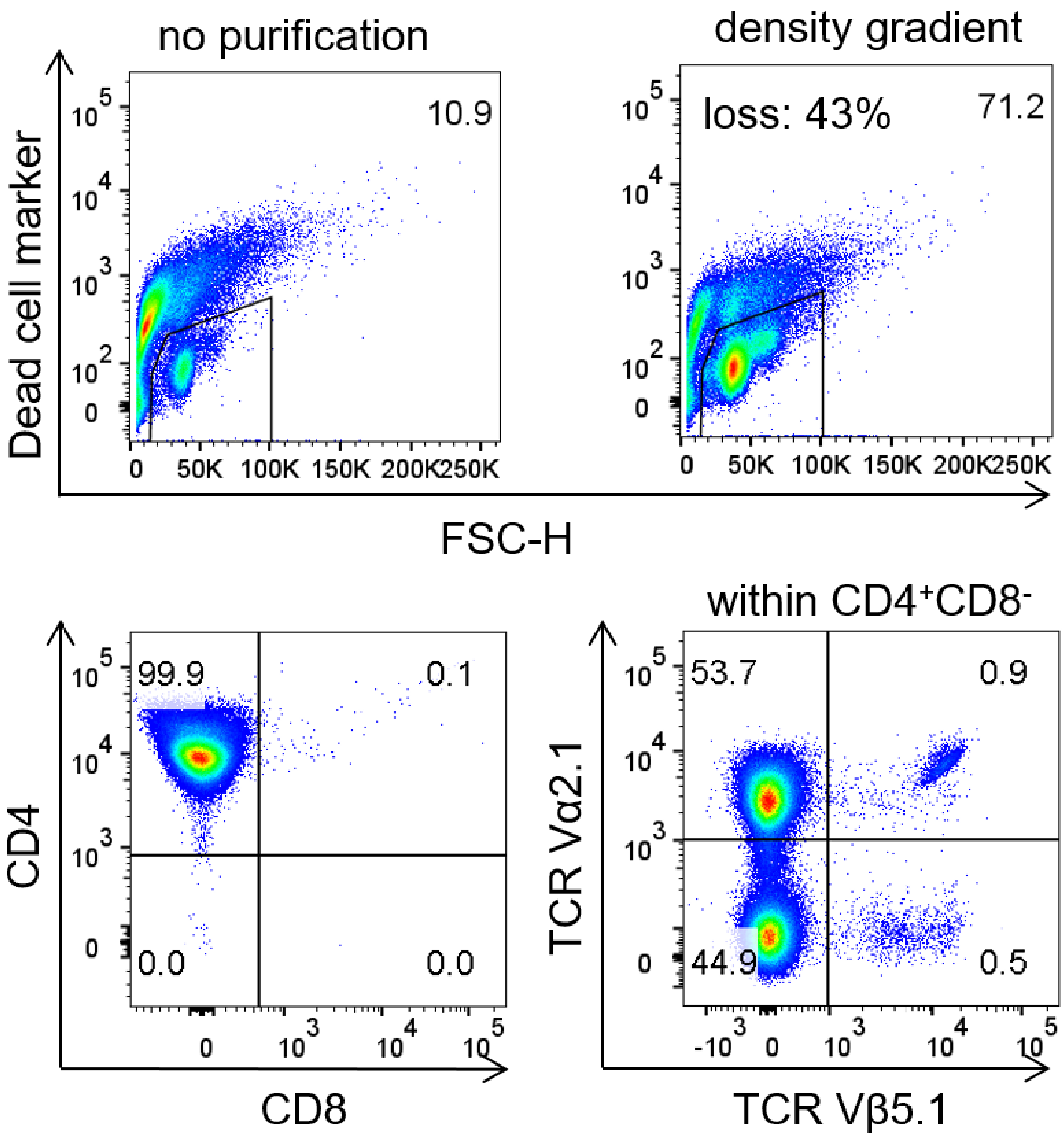

Figure S4. The established OVA$_{323\text{-}339}$-specific CD4$^+$ T cell line comprises T cells expressing various TCRs. The T cell line prior to (upper left) and after purification by density gradient centrifugation (upper right). Flow cytometric analysis revealed a pure CD4$^+$ T cell population (lower left) consisting of T cells expressing different variable regions of the TCR alpha and beta chains (lower right). Gating strategy: living cells → single cells (FSC-A vs. FSC-H) → CD4 vs. CD8 → TCR Vα2.1 vs. TCR Vβ5.1.

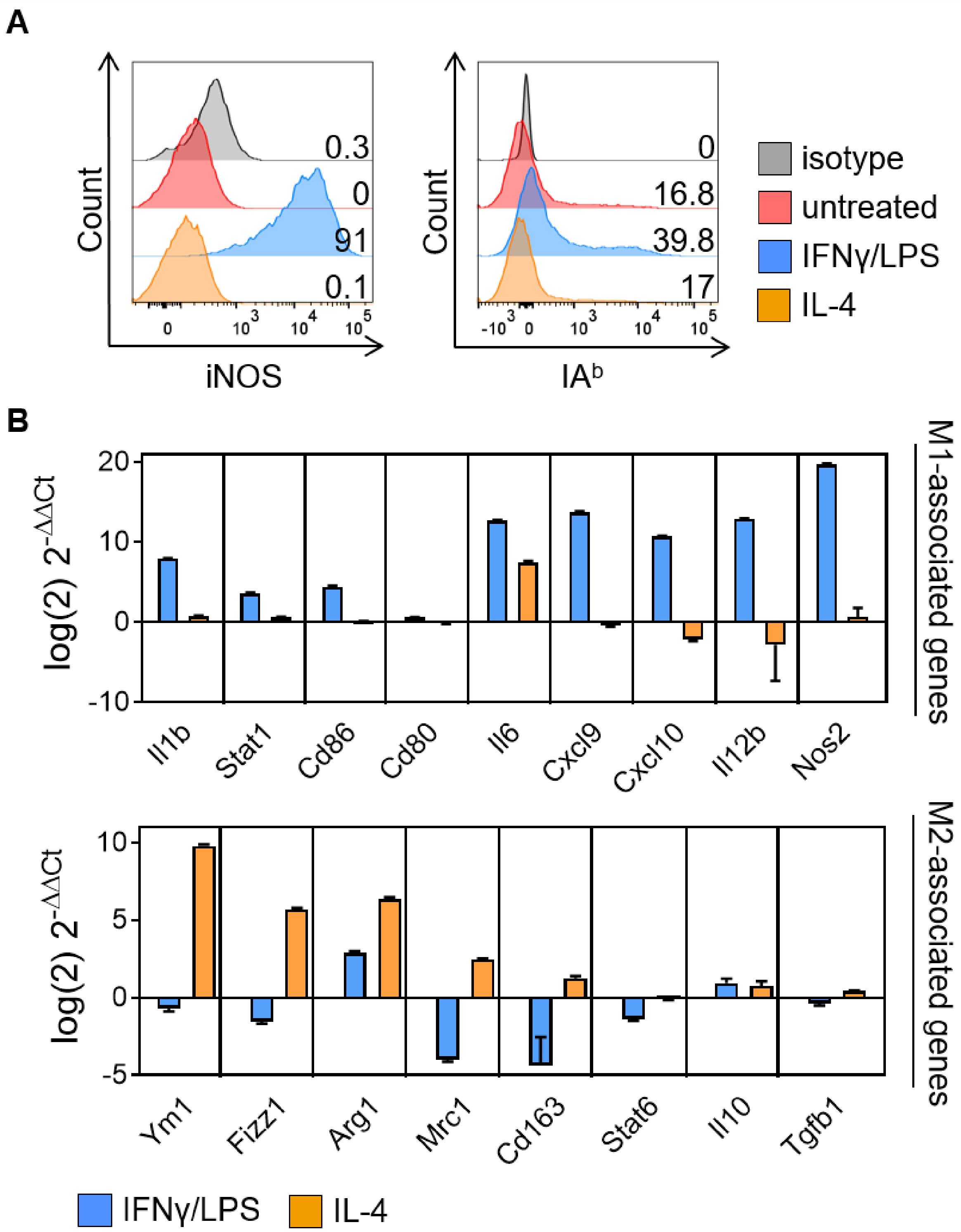

Figure S5. PECs used for repolarization by $CD4^+$ T cells display initial M2-like phenotype. PECs polarized by external cytokine addition for 24 h were tested for intracellular iNOS and IA^b surface expression by flow cytometry (**A**) as well as for TAM-associated gene expression by qPCR (**B**). Note absent iNOS and low IA^b expression as well as upregulated M2-associated gene expression of IL-4 treated PECs used for co-culture with $CD4^+$ T cells. As control, M1 polarization was induced by IFNγ/LPS treatment. Gating strategy for (A): living cells → single cells (FSC-A vs. FSC-H) → $F4/80^+CD11b^+$ → iNOS/IA^b vs. count. (B) Data obtained by external cytokine addition were normalized to untreated PECs. Representative results from one out of three experiments are shown. Error bars represent 95 % CI of technical triplicates.

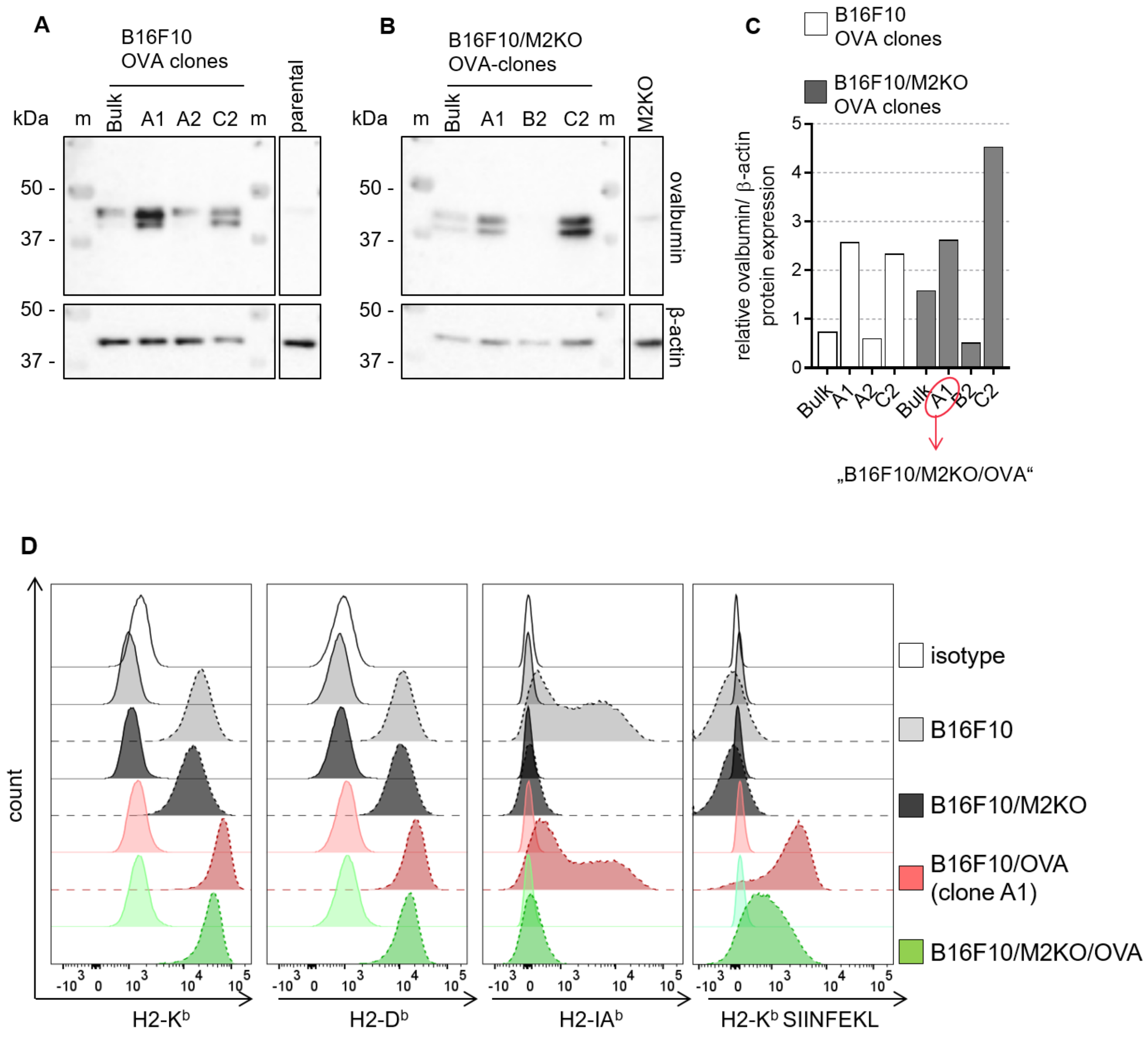

Figure S6. Phenotypic characterization of B16F10 and B16F10/M2KO cell lines transduced with OVA encoding lentivirus. OVA expression among clones derived from transduced B16F10 (**A**) and B16F10/M2KO (**B**) bulk cultures, respectively, as detected by Western blot analysis. (**C**) Quantification of OVA expression; clone B16F10/M2KO/OVA was selected for further experiments. (**D**) Expression of MHC molecules and of H2-K^{b}/SIINFEKL complexes on the cell surface of transduced clones. Cells were treated with 20 U/ml IFNγ for 48 h (dashed lines) or were left untreated (solid lines) prior to flow cytometric analysis using monoclonal antibodies against H2-K^{b}, H2-D^{b}, H2-IA^{b} and H2-K^{b}/SIINFEKL. IFNγ induced upregulation of H2-K^{b}/SIINFEKL surface expression was observed on B16F10/OVA (clone A1; red histogram) and on clone B16F10/M2KO/OVA (green histogram). No H2-K^{b}/SIINFEKL surface expression was detected on parental B16F10 and on B16F10/M2KO cells. Gating strategy: living cells → single cells (FSC-A vs. FSC-H) → marker vs. count. Antibodies used for Western blot analysis are listed in Table S1.

Supplemental Tables

Supplemental Table S1: Antibodies used for the ELISpot and Western blot assays.

Specificity	Con-jugate	Dilution	Cat. No.	Manufacturer	Clone	Isotype	Species
murine IFN-γ (1)	-	200	551216	Becton Dickinson	R4-6A2	IgG1, κ	Rat
murine IFN-γ (1)	Biotin	500	554410	Becton Dickinson	XMG1.2	IgG1, κ	Rat
murine β actin (2)	-	10000	691001	MP Biomedicals	C4	IgG1	mouse
murine IgG (2)	HRP	5000	sc-2005	Santa Cruz Biotechnology	polyclonal	ns	goat
chicken ovalbumin (2)	-	1000	sc-65984	Santa Cruz Biotechnology	3G2E1D9	IgG1	mouse

(1) ELISpot, (2) Western blot; ns: not specified

Supplemental Table S2: Monoclonal antibodies used for immunofluorescence staining and flow cytometry.

Index	Specificity	Conjugate	Cat. No.	Manufacturer	Clone	Isotype Index
101	CD11b	PerCP-Cy 5.5	101228	BioLegend	M1/70	100
112	CD206	Brilliant Violet 605	141721	BioLegend	C068C2	111
201	CD206	PE-Cy7	141719	BioLegend	C068C2	87
110	CD4	V450	560468	Becton Dickinson	RM4-5	148
53	CD45	PE	553081	Becton Dickinson	30-F11	158
154	CD45.1	APC	110714	BioLegend	A20	155
99	CD45.2	Alexa Fluor 488	109816	BioLegend	104	98
89	CD8	PE-Cy7	100722	BioLegend	53-6.7	87
145	Egr2	APC	17-6691-80	ebioscience	erongr2	146
107	F4/80	Alexa Fluor 647	123122	BioLegend	BM8	106
156	F4/80	PE-Cy7	123114	BioLegend	BM8	87
94	Gr-1	Alexa Fluor 700	108422	BioLegend	RB6-8C5	93
202	Gr-1	Alexa Fluor 488	108417	BioLegend	RB8-8C5	203
144	H2-Db	PerCP-Cy 5.5	111517	BioLegend	KH95	150
143	H2-Kb	FITC	116505	BioLegend	AF6-88.5	151

152	H-2Kb SIINFEKL	PE-Cy7	141607	BioLegend	25-D1.16	153
104	I-A[b]	Alexa Fluor 647	116412	BioLegend	AF6-120.1	103
88	NOS2	PE-Cy7	25-5920-82	eBioscience	CXNFT	87
42	TCR Vα2	FITC	127805	BioLegend	B20.1	-
43	TCR Vβ5.1	APC	139505	BioLegend	MR9-4	198

Isotype controls

Index	Specificity	Conjugate	Cat. No.	Manufacturer	Clone
87	Isotype Ctrl.	PE-Cy7	400522	BioLegend	RTK2758
93	Isotype Ctrl.	Alexa Fluor 700	400628	BioLegend	RTK4530
98	Isotype Ctrl.	Alexa Fluor 488	400233	BioLegend	MOPC-173
100	Isotype Ctrl.	PerCP-Cy 5.5	400632	BioLegend	RTK4530
103	Isotype Ctrl.	Alexa Fluor 647	400234	BioLegend	MOPC-173
106	Isotype Ctrl.	Alexa Fluor 647	400526	BioLegend	RTK2758
111	Isotype Ctrl.	Brilliant Violet 605	400539	BioLegend	RTK2758
146	Isotype Ctrl.	APC	17-4321-41	ebioscience	eBR2a
148	Isotype Ctrl.	V450	560377	Becton Dickinson	R35-95
150	Isotype Ctrl.	PerCP-Cy 5.5	400337	BioLegend	MPC-11
151	Isotype Ctrl.	FITC	400209	BioLegend	MOPC-173
153	Isotype Ctrl.	PE-Cy7	400125	BioLegend	MOPC-21
155	Isotype Ctrl.	APC	400219	BioLegend	MOPC-173
158	Isotype Ctrl.	PE	553989	Becton Dickinson	A95-1
198	Isotype Ctrl	APC	17-4714-81	ebioscience	P3.6.2.8.1
203	Isotype Ctrl.	Alexa Fluor 488	400625	BioLegend	RTK4530

Supplemental Table S3: Primers used for real-time PCR

Target	Primer	Sequence (5'-3')	Product size [bp]	Efficiency(*)	Source
Ym1	Ym1_qPCR_FP1	CACCATGGCCAAGCTCATTCTTGT	114	97.23	Tatano *et al.* 2014 [1]
	Ym1_qPCR_RP2	TATTGGCCTGTCCTTAGCCCAACT			
Fizz1	Fizz1_qPCR_FP3	ACTGCCTGTGCTTACTCGTTGACT	82	100.08	
	Fizz1_qPCR_RP4	AAAGCTGGGTTCTCCACCTCTTCA			
Cd163	CD163_fw35	TCCACACGTCCAGAACAGTC	107	83.98	
	CD163_rev36	CCTTGGAAACAGAGACAGGC			
Il6	IL-6_qPCR_FP5	GTCTTCTGGAGTACCATAGC	368	81.02	Movahedi *et al.* 2010 [2]
	IL-6_qPCR_RP6	GTCAGATACCTGACAACAGG			
Cxcl10	CXCL10_qPCR_FP7	TCTGAGTCCTCGCTCAAGTG	228	92.34	
	CXCL10_qPCR_RP8	CCTTGGGAAGATGGTGGTTA			
Arg1	ARG1_qPCR_FP19	TCACCTGAGCTTTGATGTCG	257	83.29	
	ARG1_qPCR_RP20	TTATGGTTACCCTCCCGTTG			
Mrc1	CD206_qPCR_FP23	TTGGACGGATAGATGGAGGG	182	96.84	Zhu *et al.* 2014 [3]
	CD206_qPCR_RP24	CCAGGCAGTTGAGGAGGTTC			
Il10	IL10_fw31	GCTCTTACTGACTGGCATGAG	105	111.66	Shaul *et al.* 2010 [4]
	IL10_rev32	CGCAGCTCTAGGAGCATGTG			
Stat6	STAT6_fw37	CTGGGGTGGTTTCCTCTTG	94	112.03	
	STAT6_rev38	TGCCCGGTCTCACCTAACTA			
Il1b	IL1β_fw39	CTGGTGTGTGACGTTCCCATTA	76	90.25	
	IL1β_rev40	CCGACAGCACGAGGCTTT			
Stat1	STAT1_fw41	CTGAATATTTCCCTCCTGGG	103	96.06	
	STAT1_rev42	TCCCGTACAGATGTCCATGAT			
Cd86	CD86_fw45	TCTCCACGGAAACAGCATCT	100	95.29	
	CD86_rev46	CTTACGGAAGCACCCATGAT			

Cd80	CD80_fw47	GGCAAGGCAGCAATACCTTA	94	104.43	
	CD80_rev48	CTCTTTGTGCTGCTGATTCG			
Tgfb1	TGFβ1_fw49	AAGTTGGCATGGTAGCCCTT	128	93.8	
	TGFβ1_rev50	GCCCTGGATACCAACTATTGC			
Arg2	ARG_fw53	AGGAACTGGCTGAAGTGGTTA	215	100.9	
	ARG_rev54	GATGAGAAAGGAAAGTGGCTGT			
Il12b	IL12b_fw57	AGTGACATGTGGAATGGCGT	285	105.35	
	IL12b_rev58	CAGGAGTCAGGGTACTCCCA			
Actb	bactin_fw61	ACCCTAAGGCCAACCGTGA	193	91.28	
	bactin_rev62	ATGGCGTGAGGGAGAGCATA			
Rpl19	Rpl19_fw69	TACCGGGAATCCAAGAAGATTGA	89	98.13	PrimerBank ID [5-7] 226958656c3
	Rpl19_rev70	AGGATGCGCTTGTTTTTGAAC			
Nos2	Nos2_fw81	GTTCTCAGCCCAACAATACAAGA	127	90.33	PrimerBank ID [5-7] 6754872a1
	Nos2_rev82	GTGGACGGGTCGATGTCAC			
Cxcl9	CXCL9_fw83	GGAGTTCGAGGAACCCTAGTG	82	99.68	PrimerBank ID [5-7] 162287427c1
	CXCL9_rev84	GGGATTTGTAGTGGATCGTGC			

(red)	M1-like
(blue)	M2-like
(purple)	Housekeeping gene

(*)All primers used for qPCR were tested for their amplification efficiency using a standard curve that is based on four 10 fold dilutions of a cDNA sample. The efficiency was calculated using the slope of the standard curve and the following formula:
Efficiency = (10(-1/slope)-1)*100

Table S4: Peptides

Sequence	Position	Length [aa]	MHC Restriction	Protein
ISQAVHAAHAEINEAGR	323-339	17	H2-IA^b	Ovalbumin
TPPAYRPPNAPIL	128-140	13	H2-IA^b	HBV core antigen

References (Supplement)

1) Tatano Y, Shimizu T, and Tomioka H (2014) Unique macrophages different from M1/M2 macrophages inhibit T cell mitogenesis while upregulating Th17 polarization. *Sci Rep* 4: 4146.

2) Movahedi K, Laoui D, Gysemans C, Baeten M, Stange G, Van den Bossche J, Mack M, Pipeleers D, In't Veld P *et al.* (2010) Different tumor microenvironments contain functionally distinct subsets of macrophages derived from Ly6C(high) monocytes. *Cancer Res* 70: 5728-5739.

3) Zhu L, Yang T, Li L, Sun L, Hou Y, Hu X, Zhang L, Tian H, Zhao Q *et al.* (2014) TSC1 controls macrophage polarization to prevent inflammatory disease. *Nat Commun* 5: 4696.

4) Shaul ME, Bennett G, Strissel KJ, Greenberg AS, and Obin MS (2010) Dynamic, M2-like remodeling phenotypes of CD11c+ adipose tissue macrophages during high-fat diet--induced obesity in mice. *Diabetes* 59: 1171-1181.

5) Spandidos A, Wang X, Wang H, Dragnev S, Thurber T, and Seed B (2008) A comprehensive collection of experimentally validated primers for Polymerase Chain Reaction quantitation of murine transcript abundance. *BMC Genomics* 9: 633.

6) Spandidos A, Wang X, Wang H, and Seed B (2010) PrimerBank: a resource of human and mouse PCR primer pairs for gene expression detection and quantification. *Nucleic Acids Res* 38: D792-799.

7) Wang X and Seed B (2003) A PCR primer bank for quantitative gene expression analysis. *Nucleic Acids Res* 31: e154.
